# Weakened Airway Epithelial Junctions and Enhanced Neutrophil Elastase Release Contribute to Age‐Dependent Bacteremia Risk Following Pneumococcal Pneumonia

**DOI:** 10.1111/acel.14474

**Published:** 2025-01-08

**Authors:** Shuying Xu, Tianmou Zhu, Hongmei Mou, Shumin Tan, John M. Leong

**Affiliations:** ^1^ Department of Molecular Biology and Microbiology Tufts University School of Medicine Boston MA USA; ^2^ Graduate Program in Immunology Tufts Graduate School of Biomedical Sciences Boston MA USA; ^3^ Mucosal Immunology and Biology Research Center Massachusetts General Hospital Boston MA USA; ^4^ Stuart B Levy Center for the Integrated Management of Antimicrobial Resistance Tufts University Boston MA USA

**Keywords:** aging, E‐cadherin, neutrophil transmigration, pneumolysin, *Streptococcus pneumoniae*

## Abstract

*Streptococcus pneumoniae* (*Sp*; pneumococcus), the most common agent of community‐acquired pneumonia, can spread systemically, particularly in the elderly, highlighting the need for adjunctive therapies. The airway epithelial barrier defends against bacteremia and is dependent upon apical junctional complex (AJC) proteins such as E‐cadherin. After mouse lung challenge, pneumolysin (PLY), a key *Sp* virulence factor, stimulates epithelial secretion of an inflammatory eicosanoid, triggering the infiltration of polymorphonuclear leukocytes (PMNs) that secrete high levels of neutrophil elastase (NE), thus promoting epithelial damage and systemic infection. Here, pulmonary E‐cadherin staining of intratracheally (*i.t.*) inoculated mice revealed PLY‐mediated disruption of AJC independently of PMNs. Apical infection of air–liquid interface (ALI) respiratory epithelial monolayers similarly showed that PLY disrupts AJCs. This epithelial damage promoted PMN transmigration and bacterial apical‐to‐basolateral translocation, and pharmacologically fortifying epithelial barrier function diminished both barrier breach in vitro and bacteremia in vivo. E‐cadherin staining after *Sp i.t.* inoculation of > 20‐month‐old mice, or apical infection of ALI monolayers derived from these mice, revealed an age‐associated vulnerability to PLY‐mediated AJC disruption, which in turn enhanced PMN migration and bacteremia. In addition, we found that PMNs from aged mice secrete increased levels of tissue‐damaging NE. Simultaneous pharmacological inhibition of tissue‐destructive NE and fortification of pulmonary epithelial barrier function was required to reduce the level of *Sp* bacteremia in aged mice to that of young mice. This work underscores the importance of fully characterizing the multifactorial sources of age‐associated susceptibility in devising adjunctive therapies to mitigate invasive pneumococcal disease in the elderly.

## Graphical abstract

During spread of Streptococcus pneumoniae from the lung to the bloodstream, the pneumococcal pore‐forming toxin pneumolysin (PLY) disrupts apical junction complexes (AJCs) and triggers the transmigration of polymorphonuclear leukocytes (PMNs), which secrete tissue‐destructive neutrophil elastase. Compared to their young counterparts, aged respiratory epithelial AJCs are less organized and suffer greater disruption upon pneumococcal infection, and aged PMNs secrete more elastase. Fortifying AJCs and inhibiting elastase activity during experimental lung infection reduced the bacteremia risk of aged mice to that of young mice, indicating that adjunctive treatments to reduce post‐lung infection bacteremia should address age‐related changes of both PMNs and epithelium.

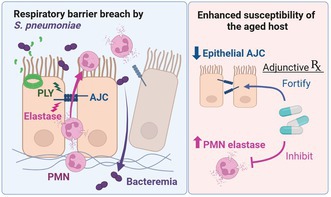

## 1 Introduction

*Streptococcus pneumoniae* (*Sp*; pneumococcus) is a common asymptomatic colonizer of the nasopharynx but can cause serious infections including pneumonia, septicemia, and meningitis (Brown et al. 2015), particularly in the elderly population (Collaborators 2018). Despite the availability of vaccines and antibiotics, *Sp* causes over 1 million deaths annually, most of which occur in individuals > 65 years of age (Collaborators 2018). Hence, complementary treatment approaches targeting detrimental age‐related changes in host response to *Sp* infection are needed, particularly those that limit complications highly associated with elderly individuals (Sundaresh et al. 2021).

A first‐line barrier against *Sp* spread is the integrity of the respiratory epithelium. Damage to the epithelium, as well as other tissues, is associated with excessive and/or sustained PMN presence in the airways (Ballinger and Standiford 2010; Burns, Abadi, and Pirofski 2005; Marks et al. 2007). The pore‐forming toxin pneumolysin (PLY) is a key *Sp* virulence factor that promotes tissue damage and invasive disease (Hirst et al. 2004; Pereira et al. 2022). We previously found that apical infection of a polarized epithelial monolayer by PLY‐producing *Sp* triggers 12‐lipoxygenase activity and production of the eicosanoid hepoxilin A3 (HXA_3_), a potent PMN chemoattractant that both promotes PMN transepithelial migration and the release of neutrophil elastase (NE) (Xu et al. 2024), a protease with tissue‐destructive potential (Boxio et al. 2016; Domon and Terao 2021; Ginzberg et al. 2001). PLY induces transmigration of tissue‐damaging PMNs that disrupts the epithelial barrier, and inhibiting HXA_3_ production or NE activity diminishes lethal bacteremia following lung infection (Bhowmick et al. 2013, 2017).

The detrimental effects of the acute inflammatory response to *Sp* lung infection are likely to be enhanced with age. In addition to age‐associated functional defects in PMN functions, such as chemotactic accuracy (Sapey et al. 2017), phagocytosis (Simell et al. 2011), and ROS production (Biasi et al. 1996), the aged host experiences inflammaging, the elevation of inflammatory signaling (Frasca and Blomberg 2016; Wenisch et al. 2000). Indeed, *Sp‐*infected elderly patients experience higher levels of PMN pulmonary influx (Menter et al. 2014; Pignatti et al. 2011), which is associated with increased bacterial burden and mortality.

These findings notwithstanding the age‐associated vulnerability to systemic *Sp* infection are complex and unlikely to be due solely to the enhanced epithelial disruption by PMNs. Epithelial barrier function is fostered by apical junctional complexes (AJCs), comprised of tight junctions (TJs) and adherens junctions (AJs) (Ganesan, Comstock, and Sajjan 2013). TJ proteins, such as zonula occludens (ZOs), claudins, occludins, and junctional adhesion molecules (JAMs), localize to the apical region of cell–cell junctions (Peter et al. 2017) and form an intercellular membrane fence (Otani and Furuse 2020). AJ proteins, such as E‐cadherin and catenins, are located basolateral to TJs and are essential for the formation and maturation of cell–cell contact (Bhatt et al. 2013). Orchestration of the abundance and localization of junction proteins, as well as their interactions with the cytoskeleton and signaling modules, are essential for maintaining optimal epithelial barrier function (Ganesan, Comstock, and Sajjan 2013). Loss of junctional integrity is associated with barrier permeability changes, and AJC malfunction of the pulmonary barrier often has deleterious outcomes during diseases of the lung, including pneumonia, acute lung injury, asthma, and chronic obstructive pulmonary disease (COPD) (Devaux, Mezouar, and Mege 2019; Ghosh et al. 2022; Nawijn et al. 2011; Wittekindt 2017).

During pulmonary infection, *Sp* targets AJC proteins for disruption and compromises function of the airway epithelial barrier (Clarke et al. 2011; LeMessurier et al. 2013; Peter et al. 2017). Pneumococcal infection reduces alveolar ZO‐1, occludins, claudins, and cadherins in human lung explants (Peter et al. 2017) and murine models (Clarke et al. 2011; Cui et al. 2024; Jacques et al. 2020; Mo et al. 2022). In addition, mice deficient in Type I interferon, which is highly susceptible to severe bacteremia following lung challenge, show junctional defects in response to *Sp* infection (LeMessurier et al. 2013). Significantly, many genes associated with the assembly of AJCs are among those with the largest age‐associated decrease in expression in the lungs (de Vries et al. 2017). Among them is *CDH1*, which encodes E‐cadherin, a key regulator of epithelial barrier function (de Vries et al. 2022; Ghosh et al. 2022; Yuksel, Ocalan, and Yilmaz 2021). E‐cadherin dysfunction promotes cell turnover and mucin accumulation, contributing to loss of barrier integrity in asthma and COPD (Ghosh et al. 2022; Kim, Schein, and Nadel 2005). Notably, we have reported that PLY promotes E‐cadherin cleavage and dissolution during *Sp* apical infection of a monolayer of immortalized respiratory cells (Xu et al. 2023). However, whether age‐related defects in AJC integrity contribute to susceptibility to *Sp* cross‐epithelial dissemination or are further exacerbated by PLY remains to be elucidated.

In this study, we *i.t*. challenged young or aged mice with *Sp*, assessing AJC disruption of the lung epithelium and subsequent bacteremia. Use of respiratory stem cell‐derived air–liquid interface (ALI) epithelial monolayers derived from young or aged mice permitted in vitro assessment of age‐related changes in epithelial AJC organization and barrier function. Additionally, ALI from mice that lack 12‐lipoxygenase activity, which is thus incapable of HXA_3_ production, enabled documentation of a role of PLY‐induced AJC disruption in *Sp*‐promoted changes in barrier function independent of PLY‐induced PMN chemoattractant secretion. Compared to lung epithelium from young mice, epithelium from aged mice was more susceptible to damage by PLY‐producing *Sp*, resulting in greater PMN transmigration, barrier disruption, and bacterial translocation. Finally, pharmacologically counteracting AJC disruption mitigated *Sp*‐induced epithelial damage and bacteremia in both young and aged mice; however, in aged mice, only the combined pharmacologic mitigation of PLY‐induced changes to both PMN behavior and epithelial AJC integrity reduced systemic spread to that of untreated young mice.

## 2 Results

### 2.1 Upon Pulmonary *Sp* Challenge of Mice, PLY Alters E‐Cadherin Organization Prior to Promoting PMN Influx, Epithelial Barrier Disruption, and Bacteremia

**FIGURE 1:**
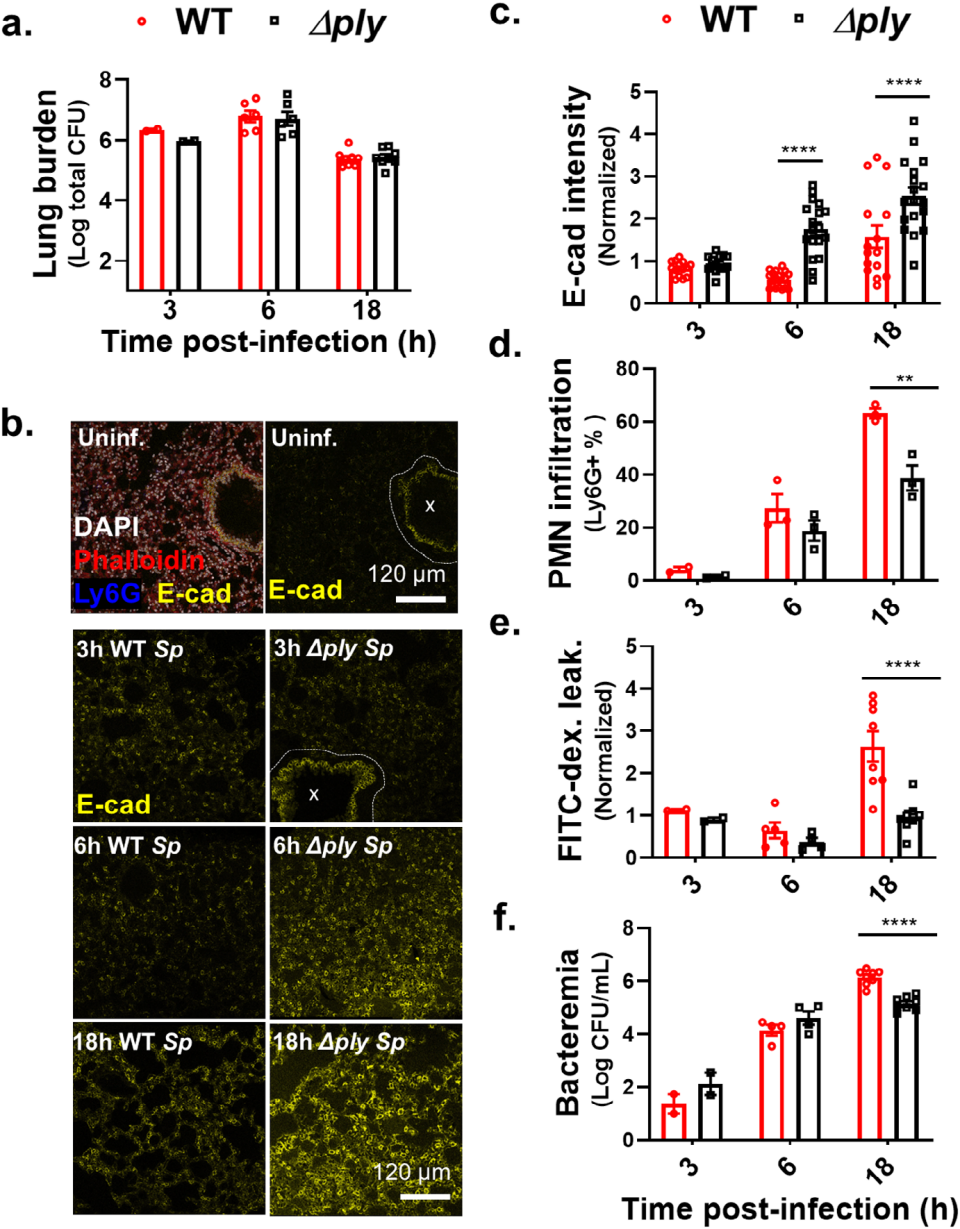
Upon pulmonary *Sp* challenge of mice, PLY alters E‐cadherin organization prior to promoting PMN influx, epithelial barrier disruption, and bacteremia. Two‐month‐old (young) BALB/c mice were infected *i.t*. with 1 × 10^7^ wild‐type (WT) or PLY‐deficient mutant (*Δply*) *Sp* for 3, 6, or 18 h. (a) Bacterial lung burden determined by measuring CFU in lung homogenates. (b) Immunofluorescence (IF) microscopy images of lung sections visualizing E‐cadherin (yellow), Ly6G (blue), nuclei (DAPI), and F‐Actin (phalloidin, red). Bronchial epithelia excluded from analysis are marked by dotted lines. (c) Alveolar E‐cadherin quantitated by signal intensity analysis in Image J, normalized to uninfected control. (d) PMN infiltration determined by flow cytometric enumeration of Ly6G^+^ cells. (e) Lung permeability quantitated by measuring the concentration of 70 kDa FITC‐dextran in the lung relative to serum after *i.v*. administration of FITC‐dextran 30 min prior to sacrifice, normalized to uninfected control. (f) Bacteremia measured by enumerating CFU in whole blood. Each panel is representative of three independent experiments, or pooled data from three independent experiments. Error bars represent mean ± SEM. Statistical analysis between WT and *Δply* groups at each time point was performed using ordinary one‐way ANOVA with Tukey's post hoc test: **p < 0.01, ****p < 0.0001.

Previously, using polarized H292 lung carcinoma cell monolayers, we found that the pore‐forming action of PLY triggers a PMN‐independent dissolution of E‐cadherin (Xu et al. 2023), a junctional protein critical for epithelial barrier function (Bryant and Stow 2004). To examine the potential role of PLY‐mediated *Sp* damage to airway epithelial junctions during pulmonary infection, we followed the kinetics of E‐cadherin disorganization, PMN infiltration, barrier disruption, and bacteremia after *i.t*. challenge of 2‐month‐old BALB/c mice with 1 × 10^7^ CFU of wild‐type (WT) or PLY‐deficient (*Δply*) *Sp*. In accord with our previous results (Xu et al. 2024), the lung burdens of WT‐ and *Δply*‐infected mice were indistinguishable during an 18‐h infection. These findings affirm that PLY had no effect on *Sp* survival in the lungs and that any PLY‐associated differences in infection parameters were independent of pulmonary load (Figure 1a).

To determine a baseline for epithelial junction integrity, we performed immunofluorescence (IF) confocal microscopy of lung sections, visualizing E‐cadherin, nuclei, F‐actin, and PMNs in uninfected animals. E‐cadherin was primarily localized to the bronchial epithelium and a few dispersed and cuboidal (likely alveolar Type II) cells along the alveolar epithelium (Figure 1b, “Uninf.”). Three hours after infection with WT or *Δply Sp*, E‐cadherin staining patterns (Figure 1b, “3 h”) and intensity were unaltered compared to uninfected lung (Figure 1c, “3 h”). Flow cytometric quantitation of pulmonary PMNs, identified by the PMN marker Ly6G, revealed that infection by either strain induced no lung inflammation at this time point (Figure 1d). Similarly, epithelial barrier function, assessed by pulmonary leakage of intravenously (*i.v*.) administered 70 kDa FITC‐dextran, was intact, and few WT or *Δply Sp* had yet escaped across the airway barrier into the bloodstream (Figure 1e,f).

Airway epithelial AJCs are upregulated in response to *Sp* invasion, a putative host‐protective response (LeMessurier et al. 2013), and at 6 h postinfection, infection with *Δply Sp* resulted in an increase in E‐cadherin staining (Figure 1b,c). In contrast, staining after infection with WT *Sp* was fourfold lower than after infection with *Δply Sp* (Figure 1b,c), indicating that PLY disrupted AJC, consistent with our previous observation that PLY promotes E‐cadherin dissolution of immortalized cell monolayers (Xu et al. 2023). Regardless of PLY production, PMNs comprised approximately 20% of cells in lung homogenates (Figure 1d) and neither strain caused detectable barrier function compromise at this time point (Figure 1e). Correspondingly, although WT and *Δply* were detected in the blood, the level of bacteremia was not influenced by the production of PLY (Figure 1f). Hence, at 6 h postinfection, PLY production did not alter acute inflammation, barrier function, or bacteremia. Rather, E‐cadherin dissolution was the sole PLY‐dependent parameter.

Finally, at 18 h postinfection, pulmonary E‐cadherin staining of WT *Sp*‐infected mice was elevated compared to uninfected mice but remained significantly lower than staining in the lungs of mice infected with *Δply Sp*, confirming that PLY promotes E‐cadherin disorganization (Figure 1b,c). In agreement with our previous work (Adams et al. 2019; Bhowmick et al. 2013), *Δply Sp* triggered less PMN pulmonary infiltration and barrier disruption (Figure 1d,e). PLY‐promoted barrier disruption at 18 h postinfection was confirmed by the PLY‐enhanced accumulation of *i.v*.‐administered FITC‐dextran in bronchoalveolar lavage fluid (BALF) infection with WT or *Δply Sp* (Figure S1a, “WT” vs. “*Δply*”). Consistent with these findings, *Δply Sp*‐infected mice displayed 10‐fold lower levels of bacteremia at 18 h postinfection compared to mice infected with WT *Sp* (Figure 1f). The lower bloodstream load of *∆ply Sp* relative to WT *Sp* was not due to diminished bloodstream fitness of the mutant because PLY deficiency did not alter bacteremia after intraperitoneal (*i.p*.) infection (Figure S1b), in agreement with previous findings that, in the TIGR4 background, PLY has no impact on bloodstream fitness (Gilley et al. 2016).

Together, these data reveal that PLY‐driven disruption to E‐cadherin (AJC) organization precedes PMN influx and epithelial barrier function disturbance.

### 2.2 PLY‐Producing *Sp* Directly Disrupt E‐Cadherin Organization and Promote PMN Transmigration and Barrier Disruption Independent of Concurrent Epithelial Cell 12‐LOX Activity

**FIGURE 2:**
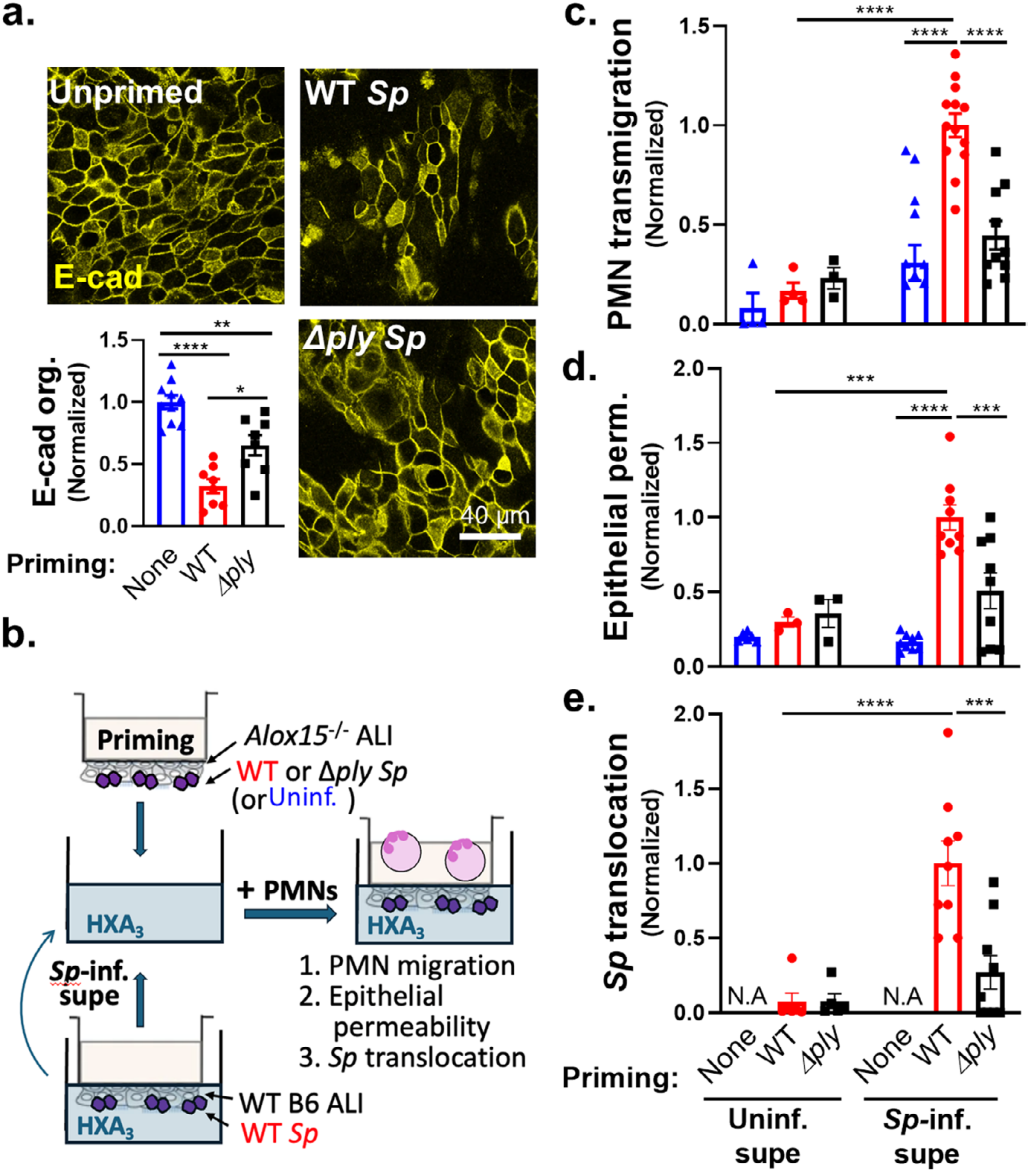
PLY‐producing *Sp* directly disrupts E‐cadherin organization and promotes PMN transmigration and barrier disruption independent of concurrent epithelial cell 12‐lipoxygenase activity. (a) Healthy adult human BSC‐derived ALI epithelial monolayers were infection primed with 1 × 10^7^ WT or *Δply Sp*. (a) IF microscopy images of fixed and permeabilized monolayers visualizing E‐cadherin localization. E‐cadherin organization quantitated by image analysis via the intercellular junction organization quotient (IJOQ) script in Python, normalized to no infection priming control. (b–e) *Alox15* ^*−/−*^ mouse‐derived ALI monolayers, which lack 12‐lipoxygenase activity and are incapable of generating HXA_3_, were infection primed with 1 × 10^7^ WT or *Δply Sp* and transferred into apical chambers containing apical supernatant harvested from uninfected or WT *Sp*‐infected WT B6 mouse‐derived ALI monolayers. 1 × 10^6^ PMNs were added basally to monolayers and allowed to migrate for 2 h. Readouts were normalized to WT *Sp*‐infection primed ALI monolayers transferred into *Sp* infection supernatant and include (b) schematic of infection priming of ALI monolayers and HXA3‐containing supernatant generation. (c) The degree of transmigration as determined by MPO activity in the apical chamber, (d) epithelial permeability measured by HRP flux, and (e) *Sp* translocation quantitated by measuring basolateral CFU. Each panel is representative of three independent experiments, or pooled data from three independent experiments. Error bars represent mean ± SEM. Statistical analyses were performed using ordinary one‐way ANOVA with Tukey's post hoc test: *p < 0.05, **p < 0.01, ***p < 0.001, ****p < 0.0001.

Epithelial monolayers derived from bronchial stem cells and grown with an ALI display many of the architectural and functional attributes of the airway mucosa, including mucus production, cilia, and AJCs that promote a robust junctional barrier (Levardon et al. 2018; Mou et al. 2016; Yonker et al. 2017). We previously utilized apical infection of murine ALI monolayers to recapitulate aspects of *Sp* pathogenesis during mucosal infection (Xu et al. 2024). To investigate PLY‐triggered AJC dissolution, we infected human‐derived ALI monolayers for 2 h with 1 × 10^7^ WT or *Δply Sp*, then visualized E‐cadherin localization by IF confocal microscopy. In uninfected ALI monolayers, E‐cadherin localized to the cell periphery, forming a connected network of circumferential rings (Figure 2a, “Unprimed.”). Upon infection, we observed a loss of this peripheral E‐cadherin that was more severe after infection by WT *Sp* than by *Δply Sp* (Figure 2a, “WT *Sp*” vs. “*Δply Sp*”), consistent with our previous work with immortalized cell monolayers (Xu et al. 2023) and the mouse infections described in Figure 1. To quantitate loss of E‐cadherin organization, we analyzed images using intercellular junction organization quotient (IJOQ), which measures the continuity in pericellular junction distribution rather than just signal intensity (Mo et al. 2022). IJOQ quantification indicated that infection by *Δply Sp* resulted in a 35% reduction in E‐cadherin organization (Figure 2a, “Priming: *Δply*”), consistent with the moderate effects of infection of immortalized monolayers by *Δply Sp* and indicative of a PLY‐independent pathway for AJC disruption (Xu et al. 2023). Infection by PLY‐producing (WT) *Sp* resulted in a significantly greater (~70%) reduction in E‐cadherin organization (Figure 2a “Priming: WT”).

The above results indicate that human ALI monolayers, like H292 monolayers (Xu et al. 2023), sustained PMN‐independent AJC injury upon infection by PLY‐producing *Sp*. This AJC disruption is not sufficient to permit apical‐to‐basolateral movement of bacteria in the absence of PMNs (Xu et al. 2024). Nevertheless, sites of AJC disorganization might be exploited as foci of PMN transmigration, analogous to “hotspots,” sites of enhanced PMN transendothelial migration (Gronloh, Arts, and van Buul 2021; Reglero‐Real et al. 2021), and we thus investigated whether PLY‐triggered AJC dissolution facilitates PMN transmigration and/or compromise of epithelial barrier function. Mouse‐derived ALI monolayers, as opposed to those derived from humans, provide a unique, tractable model for examination of this question, as the effects of PLY‐induced AJC disruption can be decoupled from PLY‐induced PMN chemoattractant secretion by utilizing ALI monolayers derived from *Alox15* ^*−/−*^ B6 mice, which are devoid of 12‐lipoxygenase activity and incapable of promoting chemotactic PMN movement (Xu et al. 2024). *Alox15* ^*−/−*^ ALI monolayers were either infected, that is, “primed,” with 1 × 10^7^ WT or *Δply Sp* to induce different degrees of AJC disorganization, or as control, left unprimed (i.e., uninfected) (Figure 2b, “Priming”). To generate HXA_3_‐containing medium, we collected supernatant from WT B6 mouse‐derived ALI cultures that had been infected with WT *Sp*, then added this (conditioned) “*Sp*‐infected” supernatant to the apical chambers of unprimed or primed *Alox15* ^*−/−*^ ALI monolayers (Figure 2b, “*Sp*‐infected supe”). Supernatant from uninfected ALI cultures served as a negative control.

1 × 10^6^ PMNs were added basally and PMN migration, epithelial permeability, and *Sp* translocation were assessed (Figure 2b). As predicted, control supernatant collected from uninfected ALI monolayers triggered no chemotactic activity, regardless of priming (Figure 2d, “Uninf. supe”). Also, as previously observed (Xu et al. 2024), *Sp*‐infected supernatant induced robust PMN transmigration across WT *Sp‐*primed *Alox15* ^*−/−*^ ALI monolayers (Figure 2d, “WT + *Sp‐*inf. supe”). Notably, transmigration across unprimed *Alox15* ^*−/−*^ ALI monolayers, which have unperturbed junctions, was minimal and not significantly greater than migration induced by uninfected supernatant (Figure 2d, “None + *Sp*‐infected supe”). Similarly, PMN migration across *Δply Sp*‐primed *Alox15* ^*−/−*^ ALI monolayers, which retained a moderate level of E‐cadherin organization (Figure 2b, “Priming: *Δply*”), was also minimal and twofold (and significantly) lower compared to the degree of transmigration across *Alox15* ^*−/−*^ ALI monolayers primed with WT *Sp* (Figure 2d, “*Δply* + *Sp‐*inf. supe”). Given that the above experiments utilized the same *Sp‐*infected supernatant to draw PMNs across the monolayers, we conclude that disruption of junctions by PLY‐producing *Sp* facilitates subsequent PMN transmigration.

In agreement with the level of PMN transmigration, when we measured barrier disruption by cross‐epithelial leakage of the protein marker horseradish peroxidase (HRP) and *Sp* translocation, only WT‐primed *Alox15* ^*−/−*^ ALI monolayers sustained significant HRP flux and *Sp* translocation after migration of PMN to HXA_3_‐containing infection supernatant (Figure 2e,f, “WT + *Sp‐*inf. supe”). Uninfected *Alox15* ^*−/−*^ ALI monolayers remained impermeable to HRP flux after PMN transmigration (Figure 2e, “Uninf. + *Sp‐*inf. supe”). *Δply‐*primed *Alox15* ^*−/−*^ ALI monolayers, which sustained less AJC injury and PMN transmigration than WT *Sp*‐primed ALI monolayers (Figure 2b,d), exhibited lower levels of HRP flux and *Sp* translocation compared to WT (Figure 2e,f, “*Δply* + *Sp‐*inf. supe”). These data suggest AJCs function as “gate keepers” of PMN influx during *Sp* infection, with PLY‐dependent junction disorganization enabling the levels of PMN transmigration that subsequently damage the epithelial barrier and promote *Sp* translocation.

### 2.3 Aged Mice Show Diminished E‐Cadherin, Increased Lung Permeability, and Increased Barrier Disruption Following Pulmonary *Sp* Challenge

**FIGURE 3:**
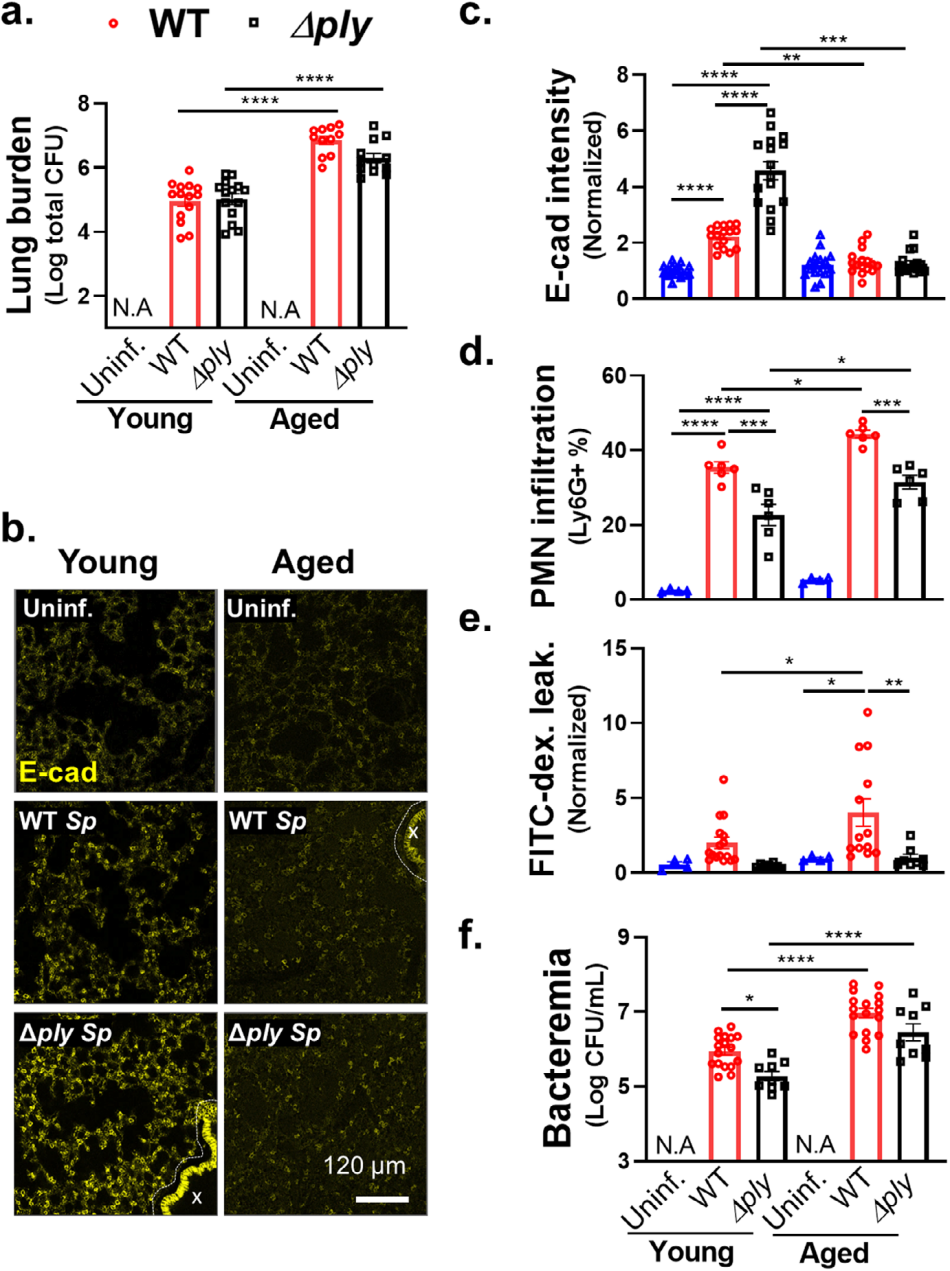
Aged mice show diminished E‐cadherin, and increased lung permeability and barrier disruption following pulmonary *Sp* challenge. Two‐month‐old (young) and 22‐month‐old (aged) BALB/c mice were infected *i.t*. with 1 × 10^7^ WT or *Δply Sp* for 18 h. (a) Bacterial lung burden determined by measuring CFU in lung homogenates. (b) Lung section IF microscopy images visualizing E‐cadherin localization. Bronchial epithelia excluded from analysis are marked by dotted lines. (c) Alveolar E‐cadherin quantitated by signal intensity analysis in Image J, normalized to uninfected control. (d) PMN infiltration determined by flow cytometric enumeration of Ly6G^+^ cells. (e) Lung permeability quantitated by measuring the concentration of 70 kDa FITC‐dextran in the lung relative to serum after *i.v*. administration of FITC‐dextran 30 min prior to sacrifice, normalized to uninfected control. (f) Bacteremia measured by enumerating CFU in whole blood. Each panel is representative of three independent experiments, or pooled data from three independent experiments. Error bars represent mean ± SEM. Statistical analyses were performed using ordinary one‐way ANOVA with Tukey's post hoc test: *p < 0.05, **p < 0.01, ***p < 0.001, ****p < 0.0001.

A decrease in TJ and AJ gene expression occurs in the aging human lung (de Vries et al. 2017), and our findings raise the possibility that age‐related changes in airway AJC integrity play a role in promoting susceptibility to *Sp* infection. We performed parallel *i.t*. infection of young (2‐month‐old) or aged (> 20‐month‐old) BALB/c mice with 1 × 10^7^ CFU of WT or *Δply Sp*. Consistent with previous reports (Bhalla et al. 2020), we found that aged mice were highly susceptible to pneumococcal infection. At 18 h.p.i., aged mice infected with WT and *Δply Sp* suffered 100‐fold and 40‐fold higher levels of lung burden than young mice, respectively (Figure 3a). Epithelial AJC integrity assessment by IF at this time point revealed lower levels of alveolar E‐cadherin in both WT and *Δply*‐infected aged mice compared to the young (Figure 3b). Image quantification indicated that while young mice exhibited robust E‐cadherin upregulation in response to *Δply Sp*, this upregulation was diminished more than twofold by the production of PLY (Figure 3c, “Young” set). In contrast, E‐cadherin levels in infected aged mice, regardless of infection by WT or *Δply Sp*, were indistinguishable from basal E‐cadherin levels of uninfected mice (Figure 3c, “Aged” set).

In accord with the lower levels of E‐cadherin in aged mice, both WT and *Δply Sp‐*infected aged mice recruited significantly more PMNs into the airways compared to their respective infections in young mice (Figure 3d). Within each set of (young or aged) infections, WT *Sp* triggers higher PMN infiltration compared to *Δply Sp* (Figure 3d), as expected given the previously established role of PLY in PMN recruitment (Adams et al. 2019; Xu et al. 2024). Also, in agreement with the lower levels of E‐cadherin and higher levels of PMN infiltration, WT *Sp* infection of aged mice was associated with FITC‐dextran leakage that was 5‐fold higher than in uninfected aged mice and 2.5‐fold higher than in young mice infected with WT *Sp* (Figure 3e, “WT”). *Δply Sp* remained unable to inflict substantial damage on the pulmonary barrier despite host age, as no change in FITC‐dextran leakage was observed in young or aged mice infected with this strain (Figure 3e, “*Δply*”). As expected from the higher lung burden, greater PMN infiltration, and more compromised lung barrier, aged mice suffered higher levels of bacteremia than the young, reflected in 5‐fold and 20‐fold higher blood burdens upon infection by WT and *Δply Sp*, respectively (Figure 3f). Together, these data show that upon *Sp* infection, mice show an age‐related decline in AJC integrity and increased PMN infiltration and bacterial spread, with a PLY‐dependent increase in pulmonary barrier leakage contributing to the exacerbation of systemic infection.

### 2.4 Age‐Related Susceptibility to E‐Cadherin Disruption and Barrier Breach During *Sp* Infection is Intrinsic to Epithelial Cells

**FIGURE 4:**
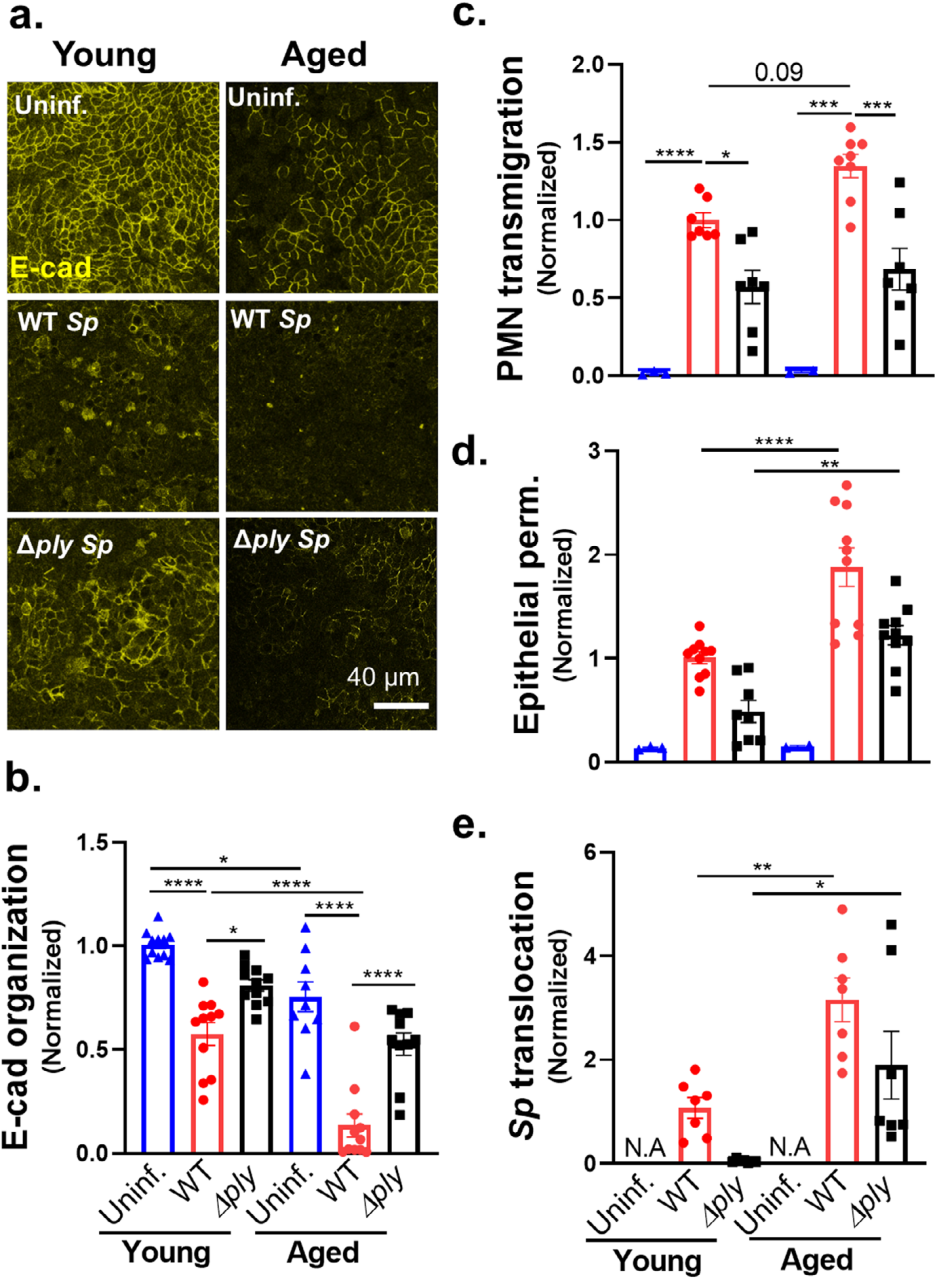
Age‐related susceptibility to E‐cadherin disruption and barrier breach during *Sp* infection is intrinsic to epithelial cells. Two‐month‐old (young) and 22‐month‐old (aged) mouse BSC‐derived ALI monolayers were apically infected with 1 × 10^7^ WT or *Δply Sp*. (a) IF microscopy images of fixed and permeabilized monolayers visualizing E‐cadherin localization. (b) E‐cadherin organization quantitated by image analysis via the IJOQ script in Python, normalized to uninfected young monolayer control. (c–e) 1 × 10^6^ PMNs were added basally to monolayers and allowed to migrate for 2 h. Readouts were normalized to WT *Sp*‐infected young monolayer and include (c) the degree of transmigration as determined by MPO activity in the apical chamber, (d) epithelial permeability measured by HRP flux, and (e) *Sp* translocation quantitated by measuring basolateral CFU. Each panel is representative of three independent experiments, or pooled data from three independent experiments. Error bars represent mean ± SEM. Statistical analyses were performed using ordinary one‐way ANOVA with Tukey's post hoc test: *p < 0.05, **p < 0.01, ***p < 0.001, and ****p < 0.0001.

To investigate whether the age‐related AJC defects observed in vivo are intrinsic to epithelial cells, we took advantage of the availability of genetically identical ALI monolayers derived from mice of different ages by isolating airway BSCs from young and aged mice to generate corresponding “young” and “aged” ALI epithelial monolayers. All ALI monolayers regardless of age passed the quality control parameter a TEER of at least 1000 Ohms and being impermeable to HRP flux before being used for infection experiments. We found that, as observed above for human ALI monolayers (Figure 2a), E‐cadherin was robustly expressed and localized to circumferential rings at cell peripheries in mouse‐derived young ALI monolayers (Figure 4a, “Young; Uninf.”). In contrast, E‐cadherin staining was weaker and less continuous in aged ALI monolayers, with IJOQ quantification showing a significant 25% reduction in E‐cadherin organization compared to young monolayers (Figure 4a,b, “Uninf.”). These data support a cell‐intrinsic nature of the weakened AJC integrity at baseline that occurs with aging (de Vries et al. 2022).

To assess the effect of aging on the epithelial response to *Sp* infection, we compared the E‐cadherin organization of young and aged ALI monolayers after parallel infections with 1 × 10^7^ WT or *Δply Sp*. By imaging and subsequent IJOQ quantitation, infection of young ALI resulted in a loss of E‐cadherin organization like that observed above in Figure 2—infection with WT and *Δply Sp* resulted in a 42% and 19% loss of E‐cadherin organization, respectively (Figure 4a,b, “Young”). Strikingly, aged ALI monolayers infected with WT *Sp* showed a near‐complete (87%) dissolution of cell peripheral E‐cadherin from an already diminished level of organization (Figure 4a,b, “Aged; WT”); *Δply Sp* infection also caused loss of E‐cadherin organization but to a lesser extent than WT *Sp* infection (Figure 4a,b, “Aged; *Δply*”). These data indicate that AJCs of aged epithelium are both less robust than AJCs of young epithelium and more prone to disruption by *Sp* infection, in accord with the results observed in vivo above.

To examine whether age‐associated epithelial AJCs defect enhanced PMN infiltration and/or *Sp* movement across the mucosal barrier, we added young PMNs to the basolateral chamber of *Sp‐*infected young or aged ALI monolayers and measured PMN transmigration, HRP flux, and *Sp* translocation. PMNs did not move across uninfected young or aged ALI monolayers (Figure 4c, “Uninf.”). Comparing young and aged ALI monolayers infected with WT *Sp*, aging appeared to result in a slight (1.3‐fold; *p* = 0.09) increase in PMN migration in response to WT *Sp* infection and was associated with significant increases in HRP flux (1.8‐fold) and *Sp* translocation (3‐fold; Figure 4c–e). Although PMN transmigration upon apical infection with *Δply Sp* was not significantly different between aged and young ALI (Figure 4c), we found that HRP flux (2.5‐fold) and *Sp* translocation (30‐fold) were both increased (Figure 4d,e). These age‐associated differences were not a result of differential secretion of chemoattractant by *Sp‐*infected young and aged ALI monolayers because supernatants collected from the respective monolayers triggered similar levels of PMN transmigration, HRP flux, and *Sp* translocation across *Alox15* ^*−/−*^ ALI monolayers (Figure S2a–c). Instead, these results suggest that epithelial cell aging, likely the age‐associated defect in AJC integrity, contributes to the increased vulnerability of aged compared to young hosts to systemic infection after *Sp* lung inoculation.

### 2.5 Junction Fortification Diminishes PMN Transmigration, Barrier Disruption, and Bacterial Translocation Upon *Sp* Infection of ALI Epithelium Derived From Young or Aged Mice

**FIGURE 5:**
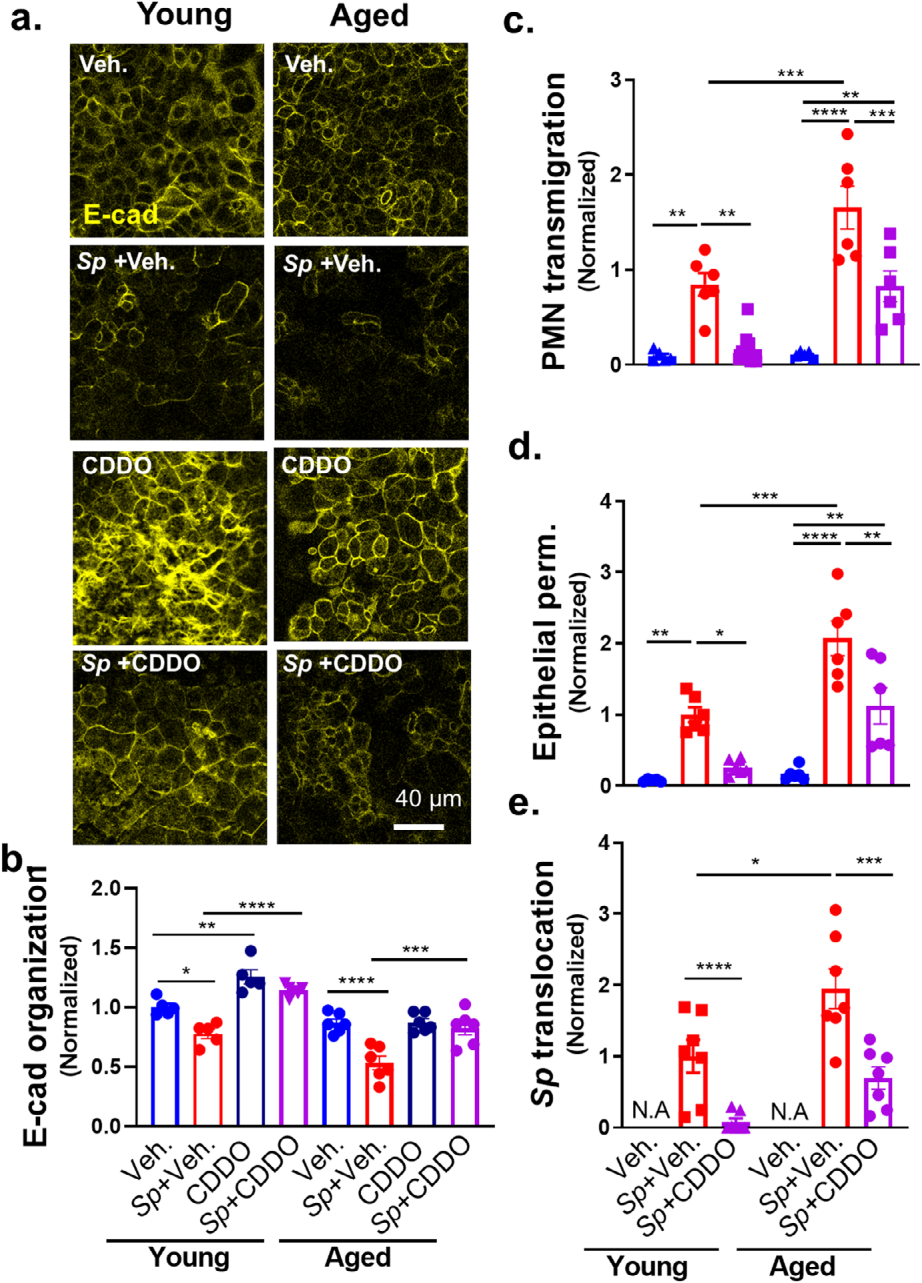
Junction fortification diminishes PMN transmigration, barrier disruption, and bacterial translocation upon *Sp* infection of ALI epithelium derived from young or aged mice. Young and aged mouse BSC‐derived ALI monolayers were pretreated with vehicle (DMSO) or 100 nM bardoxolone methyl (CDDO) before apical infection with 1 × 10^7^ WT *Sp*. (a) IF microscopy images of fixed and permeabilized monolayers visualizing E‐cadherin localization. (b) E‐cadherin organization quantitated by image analysis via the IJOQ script in Python, normalized to uninfected young monolayer control. (c–e) 1 × 10^6^ PMNs were added basally to monolayers and allowed to migrate for 2 h. Readouts were normalized to WT *Sp*‐infected, vehicle‐treated young monolayer and include (c) the degree of transmigration as determined by MPO activity in the apical chamber, (d) epithelial permeability measured by HRP flux, and (e) *Sp* translocation quantitated by measuring basolateral CFU. Each panel is representative of three independent experiments, or pooled data from three independent experiments. Error bars represent mean ± SEM. Statistical analyses were performed using ordinary one‐way ANOVA with Tukey's post hoc test: *p < 0.05, **p < 0.01, ***p < 0.001, and ****p < 0.0001.

If the age‐associated epithelial cell defect in AJC function enhances PMN transmigration, barrier compromise, and bacterial translocation upon apical *Sp* infection, these features should be mitigated by enhancing AJC integrity. Given the importance of E‐cadherin for AJC integrity (Nawijn et al. 2011; Yuksel, Ocalan, and Yilmaz 2021) and its postulated role as a key regulator of age‐related barrier defects (de Vries et al. 2022), we treated epithelial monolayers with bardoxolone methyl (CDDO), a Nrf2 agonist that promotes expression of E‐cadherin and preserves AJC function in both in vitro and mouse lung injury models (Cheng et al. 2016; Cho and Kleeberger 2015; Ghosh et al. 2022; Guo et al. 2020). Treatment with CDDO indeed enhanced E‐cadherin organization in uninfected young mouse ALI monolayers (Figure 5a,b, “Young”: “CDDO” vs. “Veh.”) and was associated with resistance to E‐cadherin dissolution upon *Sp* infection (Figure 5a,b, “Young”: “*Sp* + CDDO” vs. “*Sp* + Veh.”). Correspondingly, CDDO entirely prevented PMN transmigration, barrier compromise, and bacterial translocation upon *Sp* infection of young ALI monolayers (Figure 5c–e, “Young”: “*Sp* + CDDO” vs. “*Sp* + Veh.”).

CDDO pretreatment did not substantially improve E‐cadherin organization of uninfected aged ALI monolayers (Figure 5a,b, “Aged”: “CDDO” vs. “Veh.”). However, the E‐cadherin organization of CDDO‐treated aged ALI monolayers, like that of corresponding young ALI monolayers, was protected from *Sp*‐mediated disruption (Figure 5a,b, “Aged”: “*Sp* + CDDO” vs. “*Sp* + Veh.”). Unlike its effect on young ALI monolayers, CDDO pretreatment of aged ALI monolayers did not entirely abolish PMN transmigration, but mediated a (still significant) 40% reduction (Figure 5c; “Aged”: “*Sp* + CDDO” vs. “*Sp* + Veh.”). Correspondingly, CDDO pretreatment of aged ALI monolayers resulted in a 46% and 64% reduction in barrier integrity loss and *Sp* translocation (Figure 5d,e; “Aged”: “*Sp* + CDDO” vs. “*Sp* + Veh.”). As predicted, CDDO‐mediated AJC fortification of *Alox15* ^*−/−*^ ALI monolayers also reduced PMN transmigration, barrier disruption, and *Sp* translocation in response to *Sp*‐infected supernatant (Figure S3).

### 2.6 Barrier Disruption and *Sp* Bacteremia are Diminished by Junction Fortification and NE Inhibition in Young and Aged Mice

**FIGURE 6:**
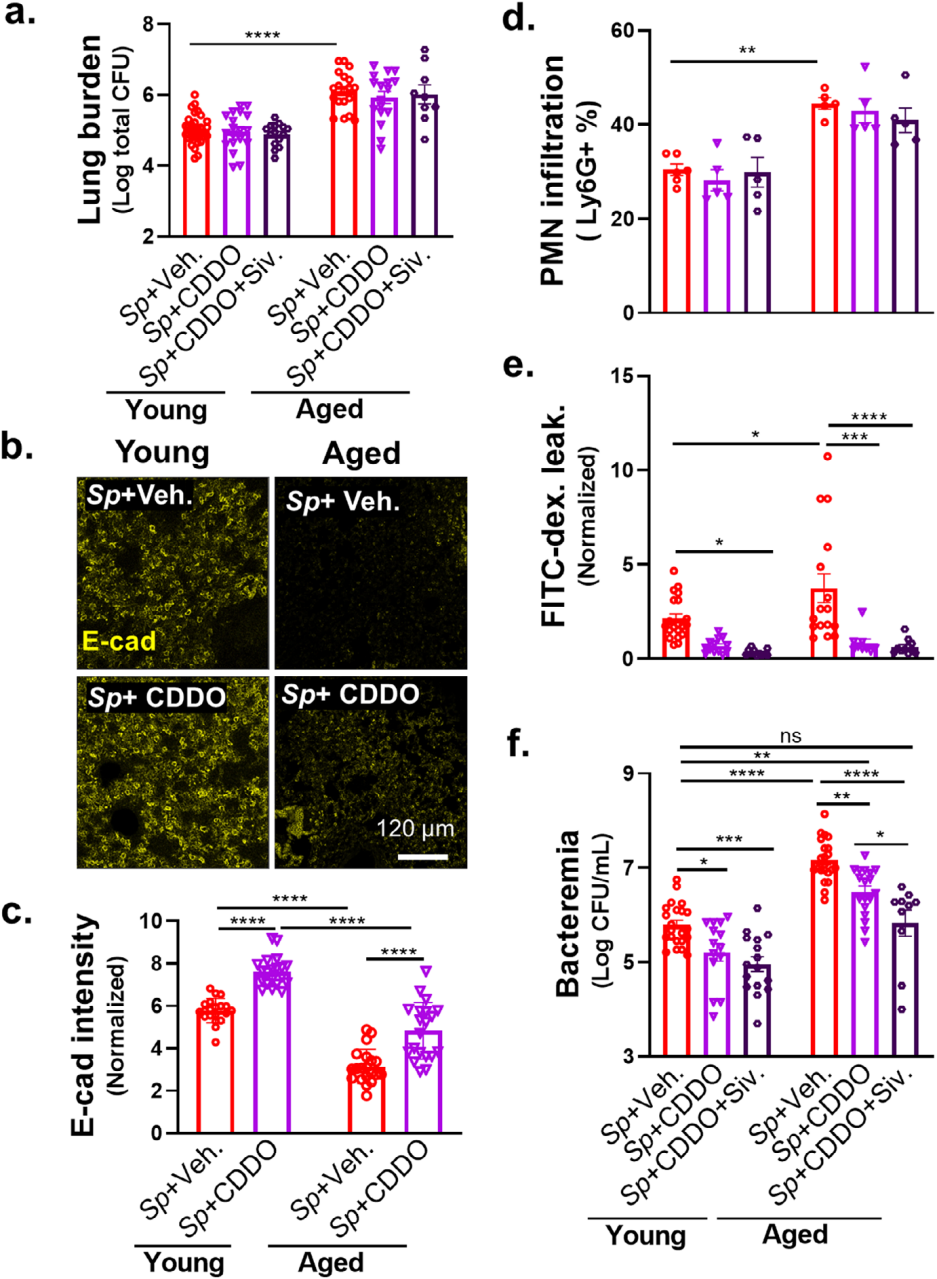
Barrier disruption and *Sp* bacteremia are diminished by junction fortification and NE inhibition in young and aged mice. Young and aged BALB/c mice were treated *i.p*. with 100 μg/mouse of CDDO or combination of 100 μg/mouse of CDDO and 500 μg/mouse sivelestat (Siv) 1 h prior to *i.t*. infection with 1 × 10^7^ WT *Sp*. (a) Bacterial lung burden determined by measuring CFU in lung homogenates. (b) Lung section IF microscopy images visualizing E‐cadherin localization. (c) Alveolar E‐cadherin quantitated by signal intensity analysis in Image J, normalized to uninfected young mouse control. (d) PMN infiltration determined by flow cytometric enumeration of Ly6G^+^ cells. (e) Lung permeability quantitated by measuring the concentration of 70 kDa FITC‐dextran in the lung relative to serum after *i.v*. administration of FITC‐dextran 30 min prior to sacrifice, normalized to uninfected young mouse control. (f) Bacteremia measured by enumerating CFU in whole blood. Each panel is representative of three independent experiments, or pooled data from three independent experiments. Error bars represent mean ± SEM. Statistical analyses were performed using ordinary one‐way ANOVA with Tukey's post hoc test: *p < 0.05, **p < 0.01, ***p < 0.001, and ****p < 0.0001.

To test whether the protective effect of CDDO on *Sp*‐mediated disruption of AJC in our mouse ALI model corresponded to protection from bacteremia after murine lung infection, we administered CDDO *i.p*. to mice 1 h before *i.t*. challenge. At 18 h.p.i, CDDO pretreatment did not alter bacterial lung burden of either young or aged mice compared to vehicle control (Figure 6a, “*Sp* + CDDO” vs. “*Sp* + Veh”), but was associated with higher E‐cadherin levels upon IF microscopy and image quantification of both young and old mice (Figure 6b,c). Notably, CDDO also did not significantly alter PMN infiltration, suggesting that the enhanced E‐cadherin did not significantly impede PMN transmigration (Figure 6d, “*Sp* + CDDO” vs. “*Sp* + Veh”). Nevertheless, CDDO pretreatment significantly decreased lung permeability to FITC‐dextran in young and aged mice by threefold and fivefold, respectively (Figure 6e, “*Sp* + CDDO” vs. “*Sp* + Veh”). We found that, in infected young mice, CDDO also significantly decreased FITC‐dextran leakage into the BALF (Figure S1b, “WT” vs. “WT + CDDO”). As predicted by its barrier‐protecting activity, CDDO diminished bacteremia by 5‐fold in the young and 10‐fold in the aged (Figure 6f).

Although CDDO pretreatment significantly diminished bacteremia in aged mice, the level of bacteremia, 3.1 × 10^6^ CFU/mL (represented in log_10_ scale in Figure 6f), was still fivefold higher (*p* < 0.01) than untreated young mice (Figure 6f, “Aged”, “*Sp* + CDDO” vs. “Young”, “*Sp* + veh.”). In our ALI monolayer model, whereas CDDO completely abrogated barrier disruption and bacterial translocation upon *Sp* infection of young ALI monolayers, its effect on these parameters of aged ALI monolayers was only partial (Figure 5d,e, “Aged” vs. “Young”; “*Sp* + CDDO” vs. “*Sp* + Veh.”), indicating that additional factors may contribute to age‐related susceptibility to barrier breach during *Sp* infection. Indeed, E‐cadherin is a substrate for NE (Ginzberg et al. 2001; Young et al. 2007), and we previously showed not only that PMNs from elderly humans exhibit elevated NE activity upon exposure to *Sp* (Bou Ghanem et al. 2017) but also that PMN NE promotes barrier disruption and bacteremia in young mice (Xu et al. 2024). Indeed, here we found that bone marrow–derived PMNs from aged mice, exposed to *Sp*, released higher levels of NE activity compared to bone marrow–derived PMNs from young mice (Figure S4a). Correspondingly, upon pulmonary *Sp* infection, higher levels of NE activity were detected in BALF from aged compared to young mice (Figure S4b).

The above results indicate that the susceptibility of the aged host to disseminated infection after lung challenge may be due to responses to *Sp* by at least two cell types, that is, aged PMNs, which secrete higher levels of NE (Bou Ghanem et al. 2017) (Figure S4), and aged lung epithelium, which exhibit compromised AJC (Figure 4). We tested whether a therapeutic strategy that addressed both defects would limit bacteremia to levels observed upon infection of untreated young mice. The NE activity inhibitor sivelestat (Siv) diminishes bacteremia following *Sp* lung challenge in young mice (Xu et al. 2024), so we subjected young and aged mice to dual CDDO and Siv treatment prior to *Sp* pulmonary challenge. In young mice, pulmonary bacterial burden, PMN infiltration, FITC‐dextran lung permeability, and bacteremia were unchanged by the addition of Siv to CDDO treatment (Figure 6a,d–f, “Young”: “*Sp* + CDDO + Siv.” vs. “*Sp* + CDDO”), suggesting that CDDO and Siv preserve AJCs in a redundant fashion in young hosts. In aged mice, the addition of Siv to CDDO treatment did not alter lung burden or the (already low) FITC‐dextran lung permeability associated with CDDO treatment alone (Figure 6a,d–f, “Aged”: “*Sp* + CDDO + Siv.” vs. “*Sp* + CDDO”). Dual treatment of aged mice was associated with only a slight (and statistically insignificant) decrease in PMN infiltration (Figure 6d, “Aged”: “*Sp* + CDDO + Siv.” vs. “*Sp* + CDDO”). However, the addition of Siv to CDDO treatment of aged mice resulted in an additional fivefold reduction in bacteremia, which, at 6.7 × 10^5^ CFU/mL, was indistinguishable from the 6.3 × 10^5^ CFU/mL observed for untreated young mice (Figure 6f, “Aged”, “*Sp* + CDDO + Siv.” vs. “Young”, “*Sp* + Veh.”). These results indicate that the combination of AJC fortification and NE blockade, by mitigating age‐associated, detrimental infection responses of both PMNs and epithelial cells, limits levels of systemic infection in aged animals to that observed in young hosts.

## 3 Discussion

A vigorous acute inflammatory response is associated with invasive pneumococcal disease (Bhowmick et al. 2013; Penaloza et al. 2015; Xu et al. 2024), so investigation of bacterial and host factors promoting excessive inflammation is likely to provide insight into the root causes of susceptibility to systemic infection by highly vulnerable populations, including the elderly. The major *Sp* virulence factor PLY (Pereira et al. 2022) can enhance pulmonary inflammation by multiple mechanisms (Gonzalez‐Juarbe et al. 2015; Parveen et al. 2024; Yoo et al. 2010). We showed that PLY triggers increased epithelial cell production of the lipid chemoattractant HXA_3_, which not only induces PMN influx but also enhances their tissue‐destructive capacity, resulting in barrier disruption and bacterial spread (Xu et al. 2023, 2024).

In addition to eliciting damaging inflammatory processes, pathogenic microbes can directly disrupt epithelial barriers to foster systemic spread (Devaux, Mezouar, and Mege 2019; Gao and Rezaee 2022; Huber 2020). Here, we showed that during mouse lung infection, PLY promoted AJC disruption independently of its effects on PMN infiltration. Using stem cell–derived ALI epithelial monolayers, which exhibit the cell types and junctional structures of *bona fide* respiratory epithelium, we recapitulated PLY‐mediated disruption of E‐cadherin organization and barrier function, consistent with our previous finding of PLY‐mediated cleavage and mislocalization of E‐cadherin in monolayers of immortalized lung epithelial cells (Xu et al. 2023). By employing ALI monolayers deficient in production of HXA_3_ (Xu et al. 2024), we were able to measure the effect of PLY on E‐cadherin disorganization independent of its effect on PMN chemoattractant production. Consistent with previous studies of PMN transmigration across epithelial barriers with disrupted AJC (Chun and Prince 2009; Morton et al. 2016), these experiments revealed that PLY‐mediated damage to AJC was required for maximal transmigration of PMNs in response to *Sp*‐induced chemoattractant. In fact, PMN transmigration in the absence of PLY‐mediated AJC disruption was both minimal and insufficient to significantly diminish barrier function. Finally, fortification of AJC by the Nrf2 agonist CDDO, which increases E‐cadherin production, diminished *Sp* translocation in ALI monolayers and bacteremia in lung‐inoculated mice. This is consistent with previous reports that Nrf2‐deficient mice are more susceptible to pneumococcal pneumonia (Gomez et al. 2016) and that Nrf2 activation limits *Sp*‐triggered epithelial cell oxidative stress (Zahlten et al. 2015). Here, we further delineate the therapeutic benefit of Nrf2 activation during *Sp* infection due to its ability to curtail PMN infiltration and thereby limit PMN‐directed epithelial barrier damage.

At baseline and during pneumococcal pneumonia, elderly patients experience elevated PMN pulmonary influx, which correlates with worse clinical outcomes (Menter et al. 2014). Aging is accompanied by a heightened state of inflammation, which alters the development of many immune effectors in response to infection (Hinojosa, Boyd, and Orihuela 2009; Meyer 2001) and is associated with elevated levels of proinflammatory cytokines and chemokines in multiple tissues, including lungs (Bouchlaka et al. 2013; Spencer et al. 1997). In addition, aging is associated with diminished epithelial barrier function (Kling et al. 2017; Thevaranjan et al. 2017). For example, lung barrier function in mice diminishes with age (Kling et al. 2017; Tankersley et al. 2003), and here, comparing the lungs of young or aged mice, we documented an age‐associated decline of E‐cadherin staining. Furthermore, an increase in E‐cadherin production in response to *Sp* lung infection was observed in young but not old mice. Elderly humans exhibit a decrease in expression of AJC genes in the lungs, including that encoding E‐cadherin (de Vries et al. 2017, 2022; Liu et al. 2024), and monolayers derived from bronchial epithelial cells from older individuals exhibit compromised barrier function (de Vries et al. 2017, 2022). Utilizing our ALI model of pulmonary epithelium derived from young or aged mice, we found that young and aged epithelium did not differ in their ability to secrete PMN chemoattractant response to *Sp* infection; rather, aging was associated with weakened AJCs that were more susceptible to *Sp*‐driven AJC damage. Mechano‐sensing triggered by PMN migration through endothelial junctions enhances PMN bactericidal activity posttransmigration (Mukhopadhyay et al. 2024) and it is tempting to speculate that the age‐associated AJC compromise and the likely concomitant decrease in PMN shear stress may diminish PMN bactericidal capacity. Finally, given that our young and aged ALI monolayers were derived from bronchial stem cells of genetically identical mice, these findings suggest that epigenetic alterations with age, a process implicated in diverse lung diseases (Hagood 2014), are responsible for the dramatic differences in monolayers derived from these stem cells derived from aged hosts.

The above insights were clearly facilitated by the experimental flexibility provided by mouse ALI monolayers. However, one limitation of the ALI model is that it reflects bronchial, not alveolar epithelium, which comprises most of the pulmonary mucosa and, due to their morphological adaptation to facilitate rapid gas exchange, form AJC comprised of TJs alone rather than both TJs and AJs as found in bronchial epithelium (Kageyama et al. 2024; Wittekindt 2017). Unfortunately, in vitro studies of *Sp* and PMN interactions with alveolar epithelium await the development of a highly physiologic alveolar monolayer model that incorporates PMNs. A second limitation of ALI modeling is that barrier function in the lung involves not just epithelial but also endothelial cells (Kageyama et al. 2024), which are not represented in our ALI monolayers. Nevertheless, PLY disrupts endothelial AJCs (Lucas et al. 2012), suggesting that *Sp* action on endothelium and epithelium may be similar. Furthermore, here we correlated findings derived from our in vitro ALI monolayer model with those from our in vivo lung infection model. In mice, disruption of the blood–airway barrier, measured by the pulmonary accumulation of *i.v*.‐delivered fluorescent dextran or the detection of bacteremia after *i.t*. inoculation, reflects translocation across both epithelial and endothelial barriers. Finally, at baseline, the epithelial barrier is approximately 10‐fold more stringent than that of endothelium, likely due to the importance of preventing penetration by airway microbes (Burns, Smith, and Walker 2003), and the concordance of conclusions from our in vitro and in vivo models suggests that during *Sp* pneumonia, the epithelium rather than the endothelium may provide the more critical barrier defense.

In addition to age‐associated defects intrinsic to epithelium, PMN defects contribute to the risk of systemic *Sp* infections. PMNs from aged mice exhibit a decline in chemotactic accuracy (Sapey et al. 2017) and antimicrobial function (Biasi et al. 1996; Simell et al. 2011), and adoptive transfer of PMNs from young mice into aged mice partially mitigates aged‐related susceptibility to *Sp* (Bhalla et al. 2020). In addition to diminished immune defense capabilities, aged PMNs exhibit activities that actively damage tissue (Van Avondt et al. 2023). For example, NE secretion by PMNs plays a key role in barrier disruption and pathogen spread during *Sp* lung infection (Xu et al. 2024), and we found here that not only do PMNs from aged mice secrete increased levels of NE ex vivo but higher levels of this protease were detected in BALF from infected aged mice.

The above findings indicate that both the decline in epithelial AJC integrity and enhanced tissue‐destructive capacity of PMNs contribute to the age‐associated susceptibility to the disseminated *Sp* infection after *i.t*. inoculation. Consistent with this, only by simultaneously targeting both age‐related immune defects by administering Siv, which inhibits NE, along with the junction‐enhancing agent CDDO, were we able to lower the level of bacteremia in aged mice to that of (untreated) young mice. The requirement for dual treatment to mitigate age‐associated risk of disseminated infection underscores the importance of fully characterizing the multifactorial sources of age‐associated susceptibility in devising adjunctive therapies to mitigate invasive pneumococcal disease in the elderly.

## 4 Materials and Methods

### 4.1 Bacterial Strains and Growth Conditions

*Sp* TIGR4 (serotype 4) and the TIGR4 PLY‐deficient mutant (*Δply*) were a gift from Dr. Andrew Camilli (Tufts University School of Medicine, MA). Bacteria were grown to mid‐exponential phase at 37°C at 5% CO_2_ in Todd Hewitt broth (BD Biosciences) supplemented with 0.5% yeast extract and oxyrase (Oxyrase, Mansfield, OH), and frozen in growth media with 20% (v/v) glycerol. Bacterial titers in aliquots were confirmed by plating serial dilutions on Tryptic Soy Agar plates supplemented with 5% sheep blood (blood agar) (Northeast Laboratory Services, Winslow, ME). For experiments, frozen aliquots were grown in liquid culture and used at mid‐log to late‐log phase.

### 4.2 Murine Infections

Young (2‐month‐old) BALB/c, C57BL/6J, and *Alox12/15* knockout (*Alox15* ^*−/−*^) (B6.129S2‐*ALOX15* ^tm1Fun^/J) mice were obtained from Jackson Laboratories. Aged (> 20‐month‐old) BALB/c and C57BL/6J mice were obtained from the National Institute on Aging aged rodent colonies. All animal experiments were performed in accordance with Tufts University Animal Care and Use Committee–approved protocols. Bedding transfers to minimize microbiota differences between mice of different breeding facilities were performed on cages within the same mouse strain. Roughly one‐quarter of soiled bedding was collected from each cage and the bedding from all cages was mixed in an empty sterile cage before redistribution across all cages.

To induce experimental pneumococcal pneumonia, young or aged BALB/c mice were *i.t*. challenged with 1 × 10^7^ colony‐forming units (CFU) of *Sp* in 50‐μL phosphate‐buffered saline (PBS). Control mice received PBS. The role of preserving junctional integrity during *Sp* infection was investigated by injection of either the Nrf2 agonist CDDO at 100 μg/mouse, in 3% DMSO, 3% Cremaphor EL (CrEL) in PBS alone, or in combination with the NE inhibitor Siv at 500 μg/mouse, in PBS, *i.p*. 1 h prior to infection. Mice were euthanized at 18 h.p.i. Blood was obtained by cardiac puncture. BALF was collected by washing the lungs with 1 mL PBS via a cannula. Whole lungs were then removed, and bacterial burden was enumerated by plating lung homogenate on blood agar plates.

### 4.3 Lung Barrier Integrity by Dextran and Microscopy

Mice were *i.v*. injected with 70 kDa MW FITC‐dextran at 5 mg/kg 30 min prior to euthanasia to assess lung permeability (Xu et al. 2024). Briefly, homogenized lungs or BALF was quantitated for FITC fluorescence using a Synergy H1 plate reader (BioTek) and readout was normalized to fluorescence in the serum of the same animal. To visualize airway epithelial junctions by fluorescence microscopy, lung tissues were harvested from euthanized mice, fixed in 4% paraformaldehyde, embedded in 4% agarose, and sectioned to a thickness of 250 μm with a Leica Vibratome (0.145 mm/s, 70 Hz, blade angle 5°) (Giacalone, Huang, and Tan 2021). Tissue sections were permeabilized with 0.1% Triton X‐100 in PBS plus 3% bovine serum albumin (BSA) for 2 h. Permeabilized sections were stained with anti‐E‐cadherin (24E10, Cell Signaling) and anti‐Ly6G (1A8, BD Pharmingen) antibodies overnight, followed by Alexa Fluor 514‐conjugated anti‐rabbit and Alexa Fluor 647‐conjugated anti‐rat secondary antibodies, along with DAPI and Alexa Fluor 594‐conjugated phalloidin (Invitrogen). Samples were mounted with Vectashield antifade mounting medium (Vector Laboratories) and visualized by confocal microscopy (Leica SP8).

### 4.4 Measurement of PMN Infiltration In Vivo

For flow cytometric quantitation of lung PMNs, lung tissues were digested into a single‐cell suspension as previously described (Xu et al. 2024). Cells were resuspended in cell staining buffer (Biolegend) and stained on ice for 30 min with APC‐conjugated anti‐Ly6G (clone 1A8, Biolegend) and then washed two times in cell staining buffer (Biolegend). Cells were analyzed using a FACSCalibur flow cytometer (BD Biosciences) and the fluorescence intensities of the stained cells were determined. Collected data were analyzed using FlowJo software (v10.7, BD) to determine the number of infiltrating PMNs by Ly6G^+^ gating (Figure S5).

### 4.5 Establishment of Epithelial ALI Monolayers

Using a previously published airway basal cell isolation and expansion protocol (Gonzalez‐Juarbe et al. 2017; Xu et al. 2024), healthy human bronchial basal cells harvested from donors without lung disease through the New England Organ Bank under an IRB‐approved protocol (MGH #2010P001354), young and aged C57BL/6J mouse‐derived tracheal basal cells, and *Alox15* ^−/−^ mouse tracheal basal cells were cultured in modified complete small airway epithelial growth media (SAGM) (Lonza, Cat. CC‐3118) (Mou et al. 2016). Cells isolated from a single donor were used between Passages 2 and 5 for consistency. To generate conventional upright monolayers on Transwells (Mou et al. 2016) for infection and imaging studies, the up‐facing side of 6.5 mm Transwell inserts with 0.4 μm pores (Corning product #3470) were precoated with 804 G rat bladder cell‐conditioned medium as a source of collagen before cell seeding. For studying neutrophil transepithelial migration, the inverted ALI model was adopted (Yonker et al. 2017), where Transwells with permeable (3 μm pore size) polycarbonate membrane inserts (Corning #3415) were used and the underside of the Transwells was 804 G medium coated before cell seeding. As previously described (Xu et al. 2024), each Transwell was seeded with 80 μL of airway basal cell suspension at a density of > 6000 cells/mm^2^, and cultured at ALI in PneumaCult‐ALI medium (StemCell Technology, Cat. 05001) for at least 21 days to allow for full epithelial maturation (Levardon et al. 2018). Transepithelial electrical resistance was assessed using a voltmeter (EVOM2, Epithelial Voltohmmeter, World Precision Instruments Inc.) to ensure the establishment of a polarized epithelial barrier.

### 4.6 Infection of ALI Monolayers

Apical surface of the ALI monolayers was infected with *Sp* at 1 × 10^7^ CFU in 25 μL of Hanks' balanced salt solution (HBSS) supplemented with 1.2 mM Ca^2+^ and 0.5 mM Mg^2+^, and incubated at 37°C with 5% CO_2_ for 2 h to allow for attachment and infection of the ALI monolayers. After treatment, Transwells were placed in 24‐well receiving plates containing HBSS with Ca^2+^ and Mg^2+^, to allow for bacteria translocation for an additional 2 h with or without the addition of 1 × 10^6^ PMNs to the basolateral chamber. 3,3′, 5,5′ Tetramethylbenzidine dihydrochloride (TMB) peroxidase substrate conversion was used to detect flux of basally added HRP to the apical chamber, as an assessment of ALI monolayer barrier integrity posttreatment. Buffer in the basolateral chambers was sampled and bacterial translocation across ALI monolayers was evaluated by plating serial dilutions on blood agar plates.

### 4.7 PMN Transepithelial Migration Assays

Whole blood obtained from healthy human volunteers under an IRB‐approved protocol (Tufts University protocol #10489) was used to isolate neutrophils using the Easysep direct human neutrophil isolation kit (Stemcell). 1 × 10^6^ PMNs were added to the basolateral chamber after 2 h of apical infection of the ALI monolayers with *Sp*.

After 2 h of transmigration, PMNs in the apical chamber were quantified by MPO activity assay (Adams et al. 2019). Briefly, transmigrated PMNs were lysed by adding 50 μL of 10% Triton X‐100 and 50 μL of 1 M citrate buffer, and lysate was transferred to a 96‐well plate. 100 μL of freshly prepared 2,2′‐azinobis‐3‐ethylbenzotiazoline‐6‐sulfonic acid (ABTS) with hydrogen peroxide solution was added to each well and incubated in the dark at room temperature for 5–10 min. Absorbance at a wavelength of 405 nm was read on a Synergy HT microplate reader (BioTek) and measurement was converted to neutrophil number using a standard curve.

### 4.8 Generation of HXA_3_ ‐Containing Supernatants to Assess the Effect of AJC Disruption (“Infection Priming”) of ALI Monolayers on PMN Migration and Bacterial Translocation

To assess the role of PLY‐induced AJC disruption independent of PLY‐induced PMN chemoattractant secretion in barrier function changes upon *Sp* infection, we first generated HXA_3_‐containing supernatants by infecting a set of young (2‐month‐old) or aged (> 20‐month‐old) WT C57BL/6J mouse basal stem cell–derived ALI monolayers with 1 × 10^7^ WT *Sp* for 1 h at 37°C with 5% CO_2_, as described by Xu et al. (2024). Infected Transwells were placed into 24‐well receiving plates containing HBSS with Ca^2+^ and Mg^2+^ in the apical chamber for an additional 1 h to allow for polarized chemoattractant secretion. At the end of incubations, apical chamber supernatants, which contain HXA_3_, were collected, centrifuged to remove residual *Sp*, and transferred to new 24‐well plates.

To generate ALI monolayers that have been subjected to AJC disruption (“primed”) by infection with *Sp*, we utilized ALI monolayers derived from *Alox15* ^*−/−*^ mice, which lack 12‐lipoxygenase activity and are incapable of producing HXA_3_. *Sp* grown to log phase were washed and resuspended to 5 × 10^8^ CFU/ml in HBSS supplemented with Ca^2+^ and Mg^2+^. 25 μL of bacterial suspension was added to the apical surface of the *Alox15* ^*−/−*^ ALI monolayers and incubated at 37°C with 5% CO_2_ for 2 h to allow for priming of the ALI monolayers. These *Sp*‐infected *Alox15* ^−/−^ ALI monolayers were then placed into plates harboring the HXA_3_‐containing supernatants generated above for PMN transmigration and barrier integrity assessment assays (Figure 2c).

### 4.9 Fluorescence Microscopy Assessment of ALI Monolayer Integrity

The degree of AJC integrity and cell confluency of ALI monolayers on Transwell filters were assessed by fluorescence microscopy. Monolayers were fixed in 4% PFA, permeabilized with 0.1% Triton‐X 100 in PBS plus 3% BSA, and stained with anti‐E‐cadherin (24E10, Cell Signaling), followed by Alexa Fluor 488‐conjugated anti‐rabbit secondary antibody, DAPI, and Alexa Fluor 594‐conjugated phalloidin. Transwell filters were then excised and mounted in Vectashield antifade mounting medium (Vector Laboratories) for visualization with a Leica SP8 spectral confocal microscope (Leica). Quantification of E‐cadherin junction organization was carried out with the python script IJOQ as previously described (43). Quantitations were normalized to that of untreated controls.

### 4.10 NE Activity Measurements

NE activity in the soluble fraction of BALF from infected mice or PMN supernatants from 1 × 10^6^ PMNs challenged with 1 × 10^7^ CFU *Sp* was determined using a PMN Elastase Fluorometric Activity Assay Kit (Abcam), following manufacturer's instructions. The area under the curve of kinetic substrate conversion curves over 2 h was measured with a Synergy H1 plate reader (BioTek) and normalized to uninfected controls.

### 4.11 Presentation of Data and Statistical Analyses

Statistical analysis was carried out using GraphPad Prism (GraphPad Software, San Diego, CA), using ordinary one‐way ANOVA followed by Tukey's post hoc test, or an unpaired t‐test in supplemental figures. *p* values < 0.05 were considered significant in all cases. Tissue and blood bacterial burdens were log‐transformed; for all other graphs, the mean values ± SEM are shown. Due to intrinsic donor‐to‐donor variability of human PMN transmigration efficacy, experiments involving human donors were normalized before pooling individual experiments. The conclusions drawn were those found to be reproducible and statistically significant across independent experiments.

## Author Contributions

S.X. and J.M.L. designed research; S.X. and T.Z. performed experiments; H.M. and S.T. contributed new reagents and protocols; S.X. and T.Z. analyzed data; and S.X., S.T., and J.M.L drafted and edited the manuscript.

## Conflicts of Interest

The authors declare no conflicts of interest.

## Supporting information


**Figure S1.** PLY has no impact on *Sp* bloodstream survival but promotes airway epithelial barrier permeability. (a) Two‐month‐old (young) BALB/c mice were infected i.t. with 1 × 107 WT or Δply *Sp*, or treated i.p. with 100 μg/mouse of CDDO and infected i.t. with 1 × 107 WT *Sp* for 18 h. Airway epithelial permeability was quantitated by measuring the concentration of 70 kDa FITC‐dextran in the BALF relative to serum after i.v. administration of FITC dextran 30 min prior to sacrifice, normalized to uninfected control. Each panel is representative of three independent experiments. (b) Two‐month‐old (young) BALB/c mice were infected i.p. with 1 × 107 WT or Δply *Sp* for 18 h. Bacteremia was measured by enumerating CFU in whole blood. Error bars represent mean ± SEM. Statistical analyses were performed using ordinary one‐way ANOVA with Tukey's post hoc test: *p < 0.05, **p < 0.01, ***p < 0.001.

**Figure S2.** Supernatants harvested upon *Sp* infection of ALI monolayers derived from young and aged ALI monolayers trigger similar levels of PMN transmigration and barrier disruption. Alox15^−/−^ mouse‐derived ALI monolayers were infection primed with 1 × 107 WT *Sp* and transferred into apical chambers containing apical supernatant harvested from young or aged mouse‐derived ALI monolayers apically infected with 1 × 107 WT *Sp*. 1 × 106 PMNs were added basally to monolayers and allowed to migrate for 2 h. Readouts were normalized to supernatant from *Sp*‐infected young ALI monolayers and include (a) the degree of transmigration as determined by MPO activity in the apical chamber, (b) epithelial permeability measured by HRP flux, and (c) *Sp* translocation quantitated by measuring basolateral CFU. Each panel represents pooled data from three independent experiments. Error bars represent mean ± SEM.

**Figure S3.** Junction fortification protects against PMN infiltration and barrier disruption independent of concurrent epithelial cell 12‐LOX activity. Alox15^−/−^ mouse‐derived ALI monolayers were pretreated with vehicle (DMSO) or 100 nM CDDO before apical infection with 1 × 107 WT *Sp* and transferred into apical chambers containing *Sp*‐infection supernatant. 1 × 106 PMNs were added basally to monolayers and allowed to migrate for 2 h. Readouts were normalized to WT *Sp*‐infected vehicle‐treated monolayers and include (a) the degree of transmigration as determined by MPO activity in the apical chamber, (b) epithelial permeability measured by HRP flux, and (c) *Sp* translocation quantitated by measuring basolateral CFU. Each panel represents pooled data from three independent experiments. Error bars represent mean ± SEM. Statistical analyses were performed using unpaired *t*‐test: **p < 0.01 and ****p < 0.0001.

**Figure S4.** Aged PMNs are associated with higher levels of NE in response to *Sp* lung challenge. (a) 1 × 106 young or aged mouse bone marrow–isolated PMNs were infected with 1 × 107 WT *Sp* and released NE activity quantitated by substrate conversion, normalized to young. (b) Young and aged BALB/c mice were i.t. infected with 1 × 107 WT *Sp*, and released NE activity in cell‐free BALF was quantitated by substrate conversion and normalized to young mice. Each panel is representative of three independent experiments, or pooled data from three independent experiments. Error bars represent mean ± SEM. Statistical analyses were performed using unpaired *t*‐test: **p < 0.01 and ***p < 0.001.

**Figure S5.** Schematic of flow gating. Schematic for gating to distinguish debris and doublet events from lung single‐cell suspension, and gating on Ly6G+ PMN events to enumerate percentage of lung‐infiltrating PMNs.

## Data Availability

The data that support the findings of this study are available from the corresponding author upon request.
