# Supplementary figures and images for: Weakened Airway Epithelial Junctions and Enhanced Neutrophil Elastase Release Contribute to Age‐Dependent Bacteremia Risk Following Pneumococcal Pneumonia
Source: Aging Cell. 2025 Jan 8;24(5):e14474. doi: 10.1111/acel.14474 (PMC12074028; doi:10.1111/acel.14474)

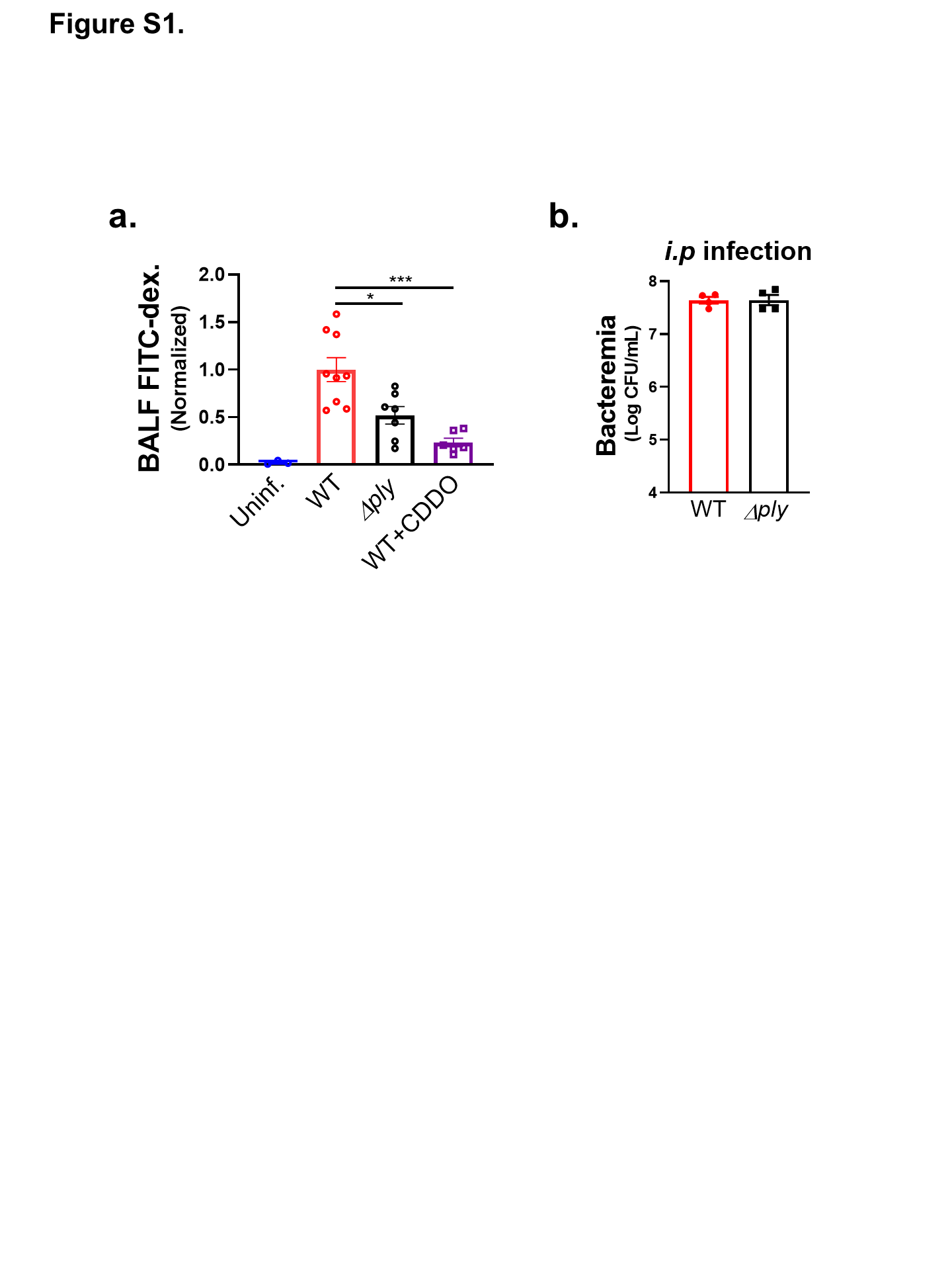

Supplement: Supplementary file 1 — Figure S1. PLY has no impact on Sp bloodstream survival but promotes airway epithelial barrier permeability. (a) Two‐month‐old (young) BALB/c mice were infected i.t. with 1 × 10^7^ WT or Δply Sp, or treated i.p. with 100 μg/mouse of CDDO and infected i.t. with 1 × 10^7^ WT Sp for 18 h. Airway epithelial permeability was quantitated by measuring the concentration of 70 kDa FITC‐dextran in the BALF relative to serum after i.v. administration of FITC dextran 30 min prior to sacrifice, normalized to uninfected control. Each panel is representative of three independent experiments. (b) Two‐month‐old (young) BALB/c mice were infected i.p. with 1 × 10^7^ WT or Δply Sp for 18 h. Bacteremia was measured by enumerating CFU in whole blood. Error bars represent mean ± SEM. Statistical analyses were performed using ordinary one‐way ANOVA with Tukey's post hoc test: *p < 0.05, **p < 0.01, ***p < 0.001. [file ACEL-24-e14474-s001.tif]

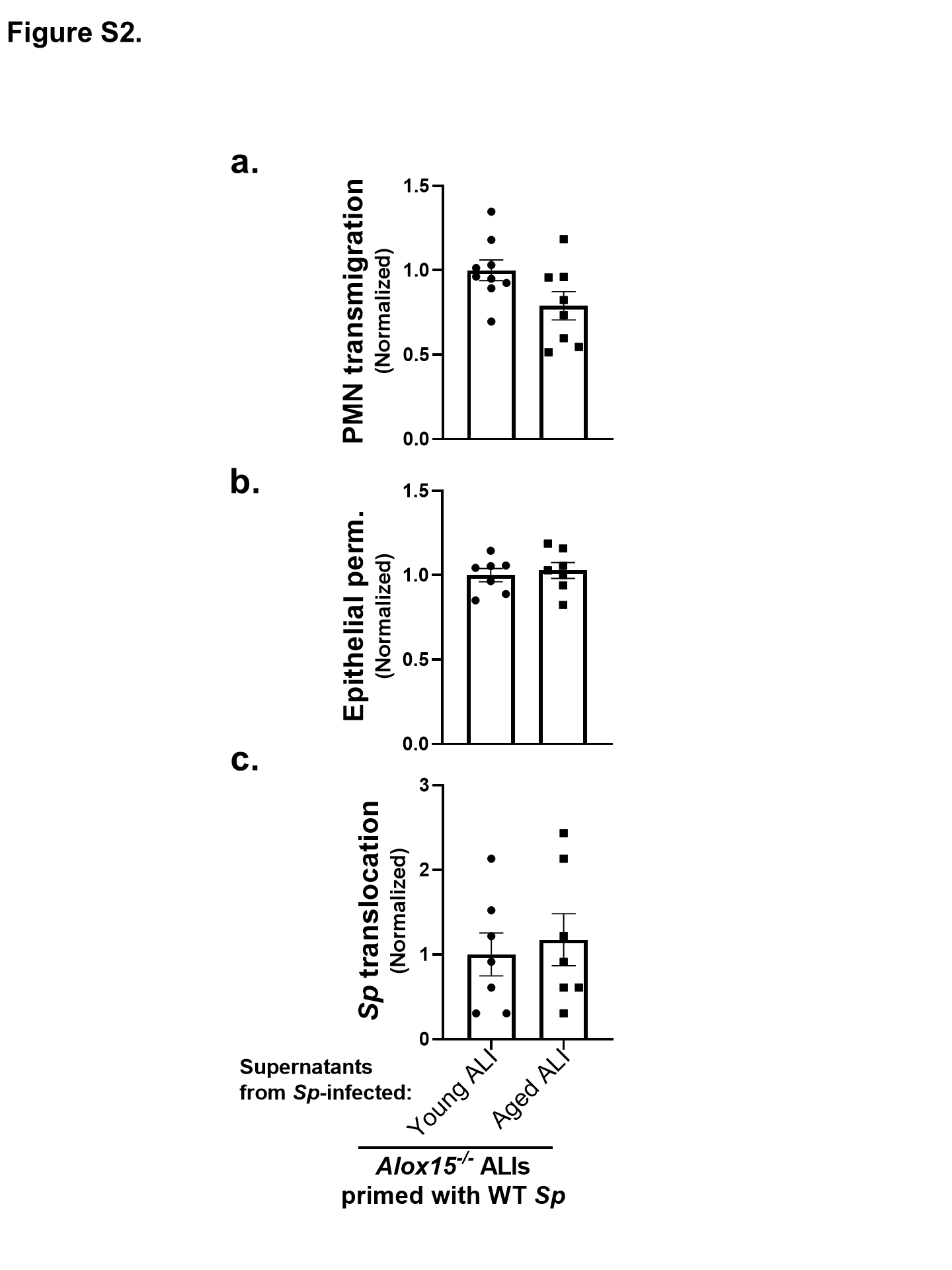

Supplement: Supplementary file 2 — Figure S2. Supernatants harvested upon Sp infection of ALI monolayers derived from young and aged ALI monolayers trigger similar levels of PMN transmigration and barrier disruption. Alox15−/− mouse‐derived ALI monolayers were infection primed with 1 × 10^7^ WT Sp and transferred into apical chambers containing apical supernatant harvested from young or aged mouse‐derived ALI monolayers apically infected with 1 × 10^7^ WT Sp. 1 × 10^6^ PMNs were added basally to monolayers and allowed to migrate for 2 h. Readouts were normalized to supernatant from Sp‐infected young ALI monolayers and include (a) the degree of transmigration as determined by MPO activity in the apical chamber, (b) epithelial permeability measured by HRP flux, and (c) Sp translocation quantitated by measuring basolateral CFU. Each panel represents pooled data from three independent experiments. Error bars represent mean ± SEM. [file ACEL-24-e14474-s005.tif]

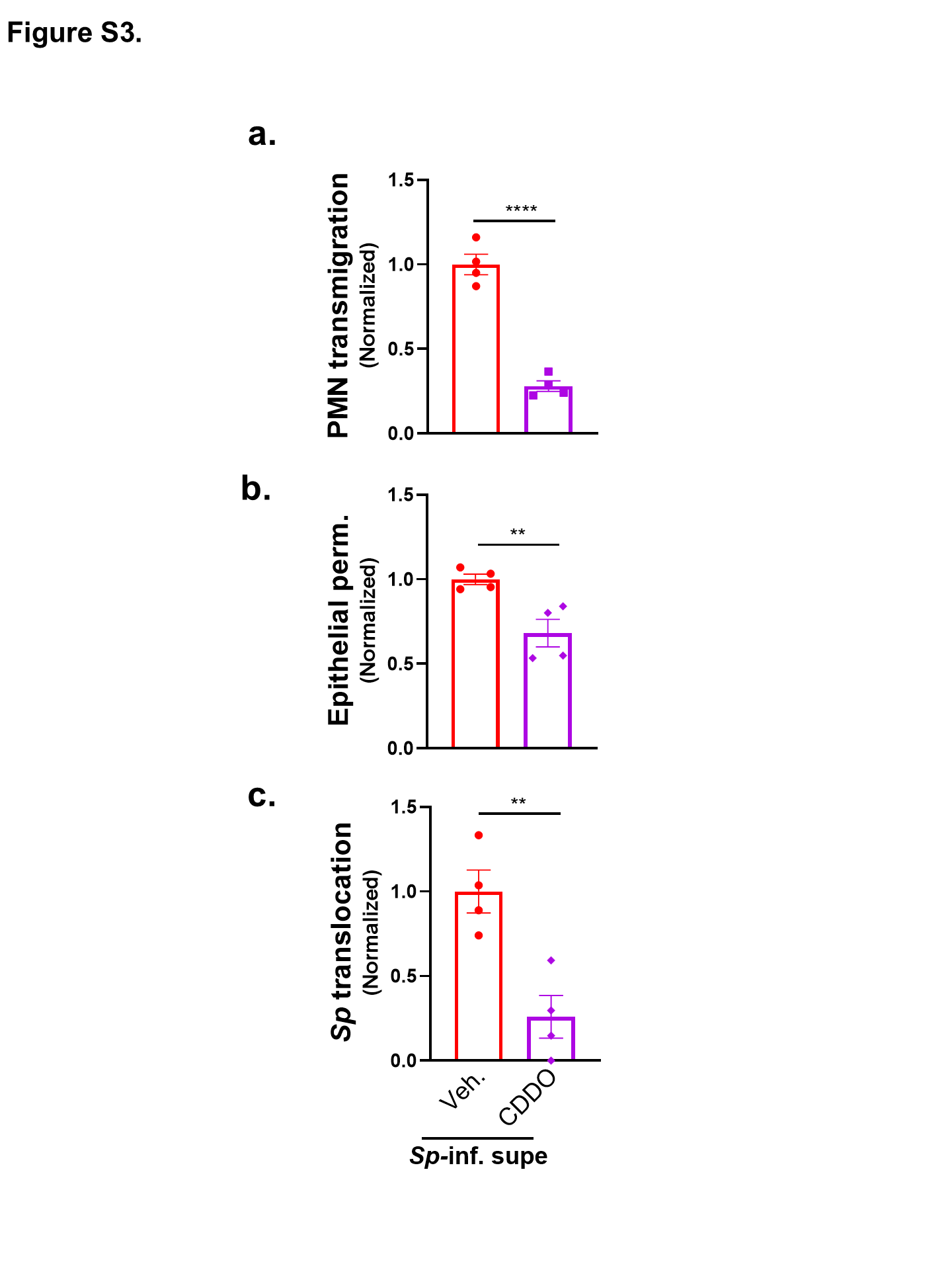

Supplement: Supplementary file 3 — Figure S3. Junction fortification protects against PMN infiltration and barrier disruption independent of concurrent epithelial cell 12‐LOX activity. Alox15−/− mouse‐derived ALI monolayers were pretreated with vehicle (DMSO) or 100 nM CDDO before apical infection with 1 × 10^7^ WT Sp and transferred into apical chambers containing Sp‐infection supernatant. 1 × 10^6^ PMNs were added basally to monolayers and allowed to migrate for 2 h. Readouts were normalized to WT Sp‐infected vehicle‐treated monolayers and include (a) the degree of transmigration as determined by MPO activity in the apical chamber, (b) epithelial permeability measured by HRP flux, and (c) Sp translocation quantitated by measuring basolateral CFU. Each panel represents pooled data from three independent experiments. Error bars represent mean ± SEM. Statistical analyses were performed using unpaired t‐test: **p < 0.01 and ****p < 0.0001. [file ACEL-24-e14474-s004.tif]

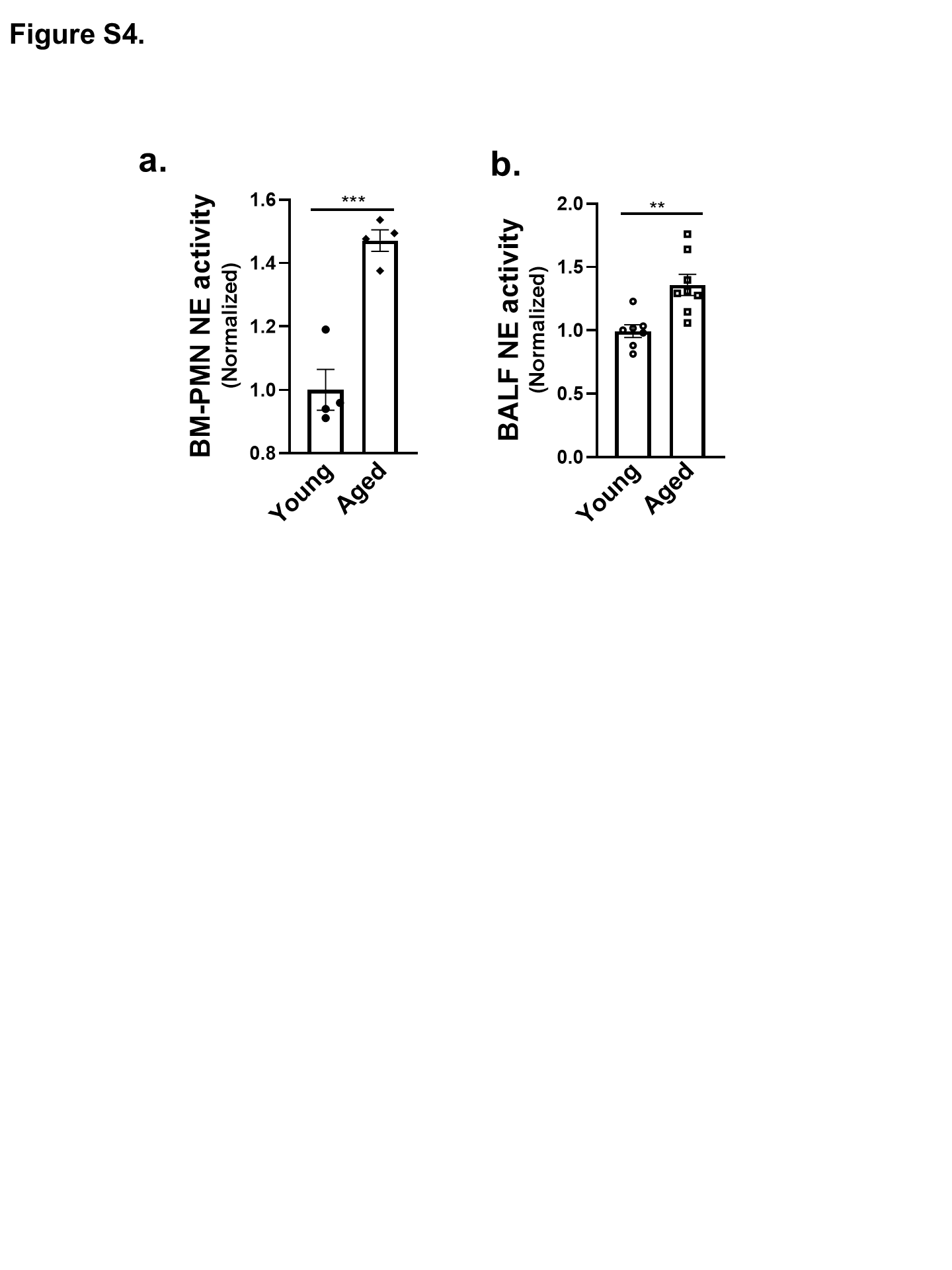

Supplement: Supplementary file 4 — Figure S4. Aged PMNs are associated with higher levels of NE in response to Sp lung challenge. (a) 1 × 10^6^ young or aged mouse bone marrow–isolated PMNs were infected with 1 × 10^7^ WT Sp and released NE activity quantitated by substrate conversion, normalized to young. (b) Young and aged BALB/c mice were i.t. infected with 1 × 10^7^ WT Sp, and released NE activity in cell‐free BALF was quantitated by substrate conversion and normalized to young mice. Each panel is representative of three independent experiments, or pooled data from three independent experiments. Error bars represent mean ± SEM. Statistical analyses were performed using unpaired t‐test: **p < 0.01 and ***p < 0.001. [file ACEL-24-e14474-s002.tif]

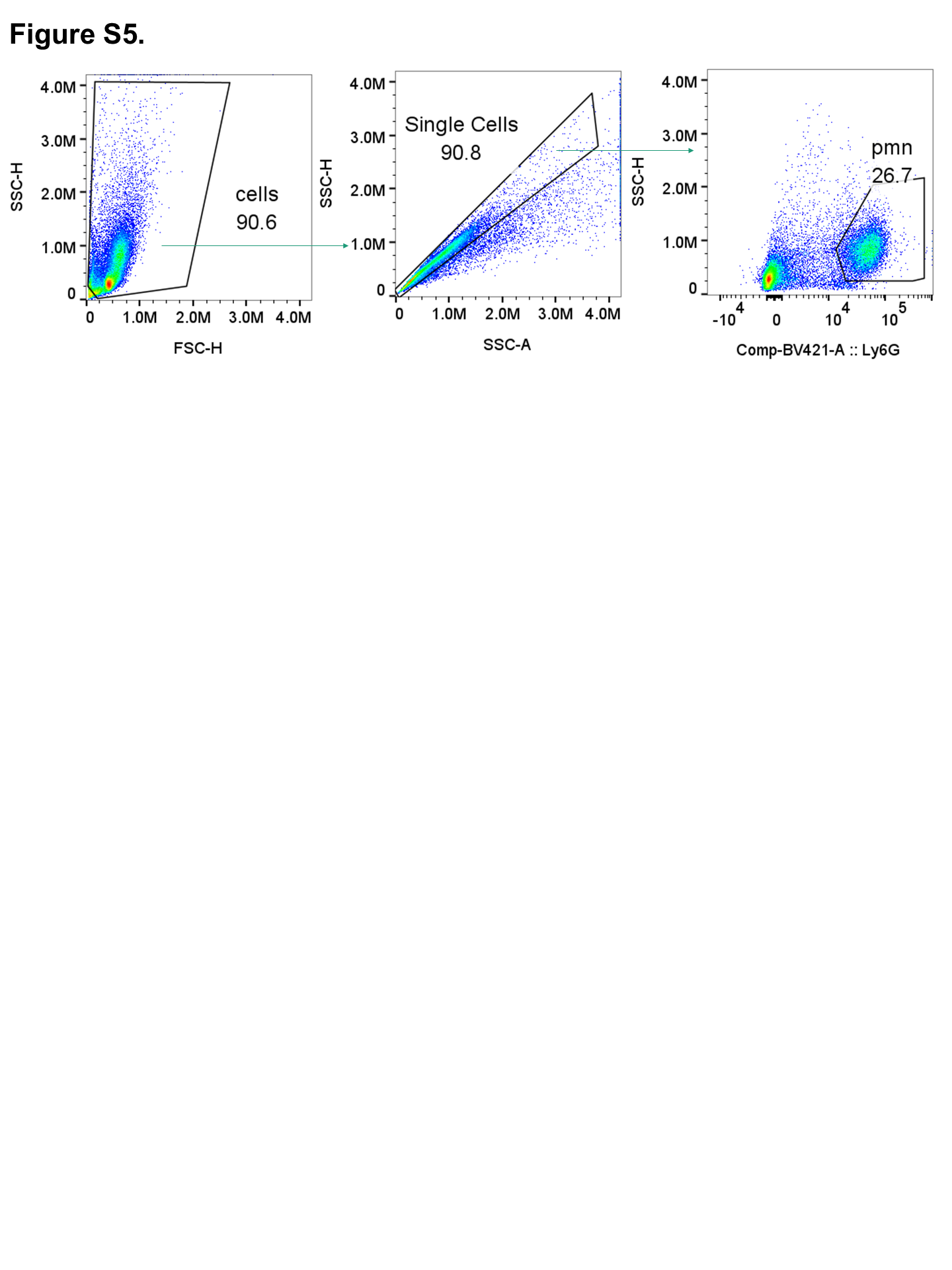

Supplement: Supplementary file 5 — Figure S5. Schematic of flow gating. Schematic for gating to distinguish debris and doublet events from lung single‐cell suspension, and gating on Ly6G+ PMN events to enumerate percentage of lung‐infiltrating PMNs. [file ACEL-24-e14474-s003.tif]
